# Rare coding variants in *RCN3* are associated with blood pressure

**DOI:** 10.1186/s12864-022-08356-4

**Published:** 2022-02-19

**Authors:** Karen Y. He, Tanika N. Kelly, Heming Wang, Jingjing Liang, Luke Zhu, Brian E. Cade, Themistocles L. Assimes, Lewis C. Becker, Amber L. Beitelshees, Lawrence F. Bielak, Adam P. Bress, Jennifer A. Brody, Yen-Pei Christy Chang, Yi-Cheng Chang, Paul S. de Vries, Ravindranath Duggirala, Ervin R. Fox, Nora Franceschini, Anna L. Furniss, Yan Gao, Xiuqing Guo, Jeffrey Haessler, Yi-Jen Hung, Shih-Jen Hwang, Marguerite Ryan Irvin, Rita R. Kalyani, Ching-Ti Liu, Chunyu Liu, Lisa Warsinger Martin, May E. Montasser, Paul M. Muntner, Stanford Mwasongwe, Take Naseri, Walter Palmas, Muagututi’a Sefuiva Reupena, Kenneth M. Rice, Wayne H.-H. Sheu, Daichi Shimbo, Jennifer A. Smith, Beverly M. Snively, Lisa R. Yanek, Wei Zhao, John Blangero, Eric Boerwinkle, Yii-Der Ida Chen, Adolfo Correa, L. Adrienne Cupples, Joanne E. Curran, Myriam Fornage, Jiang He, Lifang Hou, Robert C. Kaplan, Sharon L. R. Kardia, Eimear E. Kenny, Charles Kooperberg, Donald Lloyd-Jones, Ruth J. F. Loos, Rasika A. Mathias, Stephen T. McGarvey, Braxton D. Mitchell, Kari E. North, Patricia A. Peyser, Bruce M. Psaty, Laura M. Raffield, D. C. Rao, Susan Redline, Alex P. Reiner, Stephen S. Rich, Jerome I. Rotter, Kent D. Taylor, Russell Tracy, Ramachandran S. Vasan, The Samoan Obesity, Lifestyle and Genetic Adaptations Study (OLaGA) Group, NHLBI Trans-Omics for Precision Medicine (TOPMed) Consortium, Alanna C. Morrison, Daniel Levy, Aravinda Chakravarti, Donna K. Arnett, Xiaofeng Zhu

**Affiliations:** 1 Department of Population and Quantitative Health Sciences, Case Western Reserve University, Wolstein Research Building, 2103 Cornell Road, Cleveland, OH 44106 USA; 2 Department of Epidemiology, Tulane University School of Public Health and Tropical Medicine, New Orleans, LA USA; 3 Division of Sleep and Circadian Disorders, Brigham and Women’s Hospital, Boston, MA USA; 4 Program in Medical and Population Genetics, Broad Institute, Cambridge, MA USA; 5 Division of Sleep Medicine, Harvard Medical School, Boston, MA USA; 6 Center for Human Genetics & Genomics, New York University Grossman School of Medicine, New York, NY USA; 7 Department of Medicine (Division of Cardiovascular Medicine), Stanford University, Palo Alto, CA USA; 8 GeneSTAR Research Program, Department of Medicine, Divisions of Cardiology and General Internal Medicine, Johns Hopkins University School of Medicine, Baltimore, MD USA; 9 Division of Endocrinology, Diabetes, and Nutrition, Program for Personalized and Genomic Medicine, Department of Medicine, University of Maryland School of Medicine, Baltimore, MD USA; 10 Department of Epidemiology, School of Public Health, University of Michigan, Ann Arbor, MI USA; 11 Department of Population Health Sciences, University of Utah School of Medicine, Salt Lake City, UT USA; 12 Cardiovascular Health Research Unit, Department of Medicine, University of Washington, Seattle, WA USA; 13 Graduate Institute of Medical Genomics and Proteomics, National Taiwan University, Taipei City, Taiwan; 14 Institute of Biomedical Sciences, Academia Sinica, Taipei City, Taiwan; 15 Department of Internal Medicine, National Taiwan University Hospital, Taipei, Taiwan; 16 Human Genetics Center, Department of Epidemiology, Human Genetics, and Environmental Sciences, School of Public Health, The University of Texas Health Science Center at Houston, Houston, TX USA; 17 Department of Human Genetics and South Texas Diabetes and Obesity Institute, University of Texas Rio Grande Valley School of Medicine, Brownsville, TX USA; 18 Division of Cardiovascular Diseases, Department of Medicine, University of Mississippi Medical Center, Jackson, MS USA; 19 Department of Epidemiology, UNC Gillings School of Global Public Health, Chapel Hill, NC USA; 20 Jackson Heart Study, University of Mississippi Medical Center, Jackson, MS USA; 21 Department of Physiology and Biophysics, University of Mississippi Medical Center, Jackson, MS USA; 22 The Institute for Translational Genomics and Population Sciences, Department of Pediatrics, The Lundquist Institute for Biomedical Innovation at Harbor-UCLA Medical Center, Torrance, CA USA; 23 Division of Public Health Sciences, Fred Hutchinson Cancer Research Center, Seattle, WA USA; 24 Institute of Preventive Medicine, National Defense Medical Center, New Taipei City, Taiwan; 25 Department of Biostatistics, School of Public Health, Boston University, Boston, MA USA; 26 Framingham Heart Study, National Heart, Lung, and Blood Institute’s Framingham Heart Study, Framingham, MA USA; 27 Department of Epidemiology, University of Alabama at Birmingham, Birmingham, AB USA; 28 GeneSTAR Research Program, Department of Medicine, Division of Endocrinology, Diabetes and Metabolism, Johns Hopkins University School of Medicine, Baltimore, MD USA; 29 Division of Cardiology, Department of Medicine, George Washington University, Washington, DC USA; 30 Jackson Heart Study, Jackson State University, Jackson, MS USA; 31 Ministry of Health, Government of Samoa, Apia, Samoa; 32 Division of Cardiology, Columbia University Irving Medical Center, New York, NY USA; 33 Lutia I Puava Ae Mapu I Fagalele, Apia, Samoa; 34 Department of Biostatistics, School of Public Health, University of Washington, Seattle, WA USA; 35 Division of Endocrinology and Metabolism, Department of Internal Medicine, Taichung Veterans General Hospital, Taichung City, Taiwan; 36 Survey Research Center, Institute for Social Research, University of Michigan, Ann Arbor, MI USA; 37 Department of Biostatistics and Data Science, Wake Forest School of Medicine, Winston-Salem, NC USA; 38 GeneSTAR Research Program, Department of Medicine, Division of General Internal Medicine, Johns Hopkins University School of Medicine, Baltimore, MD USA; 39 Human Genome Sequencing Center, Baylor College of Medicine, Houston, TX USA; 40 Division of Genomic Outcomes, Department of Pediatrics, Harbor-UCLA Medical Center Professor of Pediatrics, UCLA, Torrance, CA USA; 41 Brown Foundation Institute of Molecular Medicine, McGovern Medical School, The University of Texas Health Science Center at Houston, Houston, TX USA; 42 Department of Preventive Medicine, Feinberg School of Medicine, Northwestern University Chicago, Evanston, IL USA; 43 Department of Epidemiology and Population Health, Albert Einstein College of Medicine, 1300 Morris Park Avenue, Bronx, NY USA; 44 Institute for Genomic Health, Icahn School of Medicine at Mount Sinai, New York, NY USA; 45 The Charles Bronfman Institute for Personalized Medicine, Icahn School of Medicine at Mount Sinai, New York, NY USA; 46 GeneSTAR Research Program, Department of Medicine, Divisions of Allergy and Clinical Immunology and General Internal Medicine, Johns Hopkins University School of Medicine, Baltimore, MD USA; 47 International Health Institute and Department of Epidemiology, School of Public Health, Brown University, Providence, RI USA; 48 Department of Anthropology, Brown University, Providence, RI USA; 49 Geriatrics Research and Education Clinical Center, Veterans Affairs Medical Center, Baltimore, MD USA; 50 Department of Genetics, University of North Carolina at Chapel Hill, Chapel Hill, NC USA; 51 Cardiovascular Health Research Unit, Departments of Medicine, Epidemiology, and Health Systems and Population Health, University of Washington, Seattle, WA USA; 52 Division of Biostatistics, Washington University School of Medicine, St. Louis, MO USA; 53 Department of Epidemiology, University of Washington, Seattle, WA USA; 54 Center for Public Health Genomics, University of Virginia, Charlottesville, VA USA; 55 Department of Pathology & Laboratory Medicine, Larner College of Medicine, University of Vermont, Burlington, VT USA; 56 Department of Biochemistry, University of Vermont, Burlington, VT USA; 57 Department of Medicine, School of Medicine, Boston University, Boston, MA USA; 58 Population Sciences Branch, National Heart, Lung, and Blood Institute, National Institutes of Health, Bethesda, MD USA; 59 University of Kentucky College of Public Health, Lexington, KY USA

**Keywords:** Rare variant analysis, Blood pressure, Whole genome sequencing

## Abstract

**Background:**

While large genome-wide association studies have identified nearly one thousand loci associated with variation in blood pressure, rare variant identification is still a challenge. In family-based cohorts, genome-wide linkage scans have been successful in identifying rare genetic variants for blood pressure. This study aims to identify low frequency and rare genetic variants within previously reported linkage regions on chromosomes 1 and 19 in African American families from the Trans-Omics for Precision Medicine (TOPMed) program. Genetic association analyses weighted by linkage evidence were completed with whole genome sequencing data within and across TOPMed ancestral groups consisting of 60,388 individuals of European, African, East Asian, Hispanic, and Samoan ancestries.

**Results:**

Associations of low frequency and rare variants in *RCN3* and multiple other genes were observed for blood pressure traits in TOPMed samples. The association of low frequency and rare coding variants in *RCN3* was further replicated in UK Biobank samples (*N* = 403,522), and reached genome-wide significance for diastolic blood pressure (*p* = 2.01 × 10^− 7^).

**Conclusions:**

Low frequency and rare variants in *RCN3* contributes blood pressure variation. This study demonstrates that focusing association analyses in linkage regions greatly reduces multiple-testing burden and improves power to identify novel rare variants associated with blood pressure traits.

**Supplementary Information:**

The online version contains supplementary material available at 10.1186/s12864-022-08356-4.

## Background

Compared to European Americans (EA), African Americans (AA) consistently have higher blood pressure (BP) levels with earlier onset of hypertension [1]. The excess risks from elevated blood pressure directly affect the life expectancy of AA, which is considerably lower than that of EA. Compared to their EA counterparts, AA men are twice as likely to have a stroke, with earlier onset, or develop stroke-related disabilities [2]. Despite these alarming statistics, there are few genetic studies focusing on BP traits in AA with relatively smaller sample sizes than in European-ancestry studies [3–5]. We propose that leveraging linkage evidence from family-based studies can expedite the discovery of rare variants using WGS data.

Previous studies have shown that linkage evidence could facilitate the discovery of low frequency and rare variants associated with BP or other traits [6–10]. The same approach could be applied to family-based studies with AA. A linkage analysis using 4394 AA in 1802 families from the Family Blood Pressure Program (FBPP) identified several linkage peaks on chromosomes 1, 17, and 19 (maximum logarithm of the odds [LOD] > 3) for BP traits [11]. Wang et al. have examined the 1q31 region using exome array data and have detected multiple genes and rare variants contributing to pulse pressure (PP) variation [11]. Because exome array data is limited to exonic regions with mostly coding variants, regulatory non-coding variants as well as very rare variants (minor allele frequency [MAF] < 0.001) cannot be studied with high confidence. These two challenges could be overcome with the Trans-Omics for Precision Medicine (TOPMed) whole genome sequencing (WGS) project, which surveys the whole genome and provides a target coverage of 30x on average [12]. A large number of AA families from FBPP have been whole-genome sequenced as part of TOPMed. To date, most of the large BP genetic studies have focused on samples with European ancestry, and the discovery in African ancestry falls far behind [3, 5]. TOPMed contains one of the largest samples of WGS data in AA, which makes it a suitable dataset to study rare variants found in individuals of African ancestry. In this study, we use the linkage evidence observed from AAs to guide association analysis in multiple ancestral population samples.

## Results

### Linkage analysis of AA families with TOPMed WGS data

**Fig. 1:**
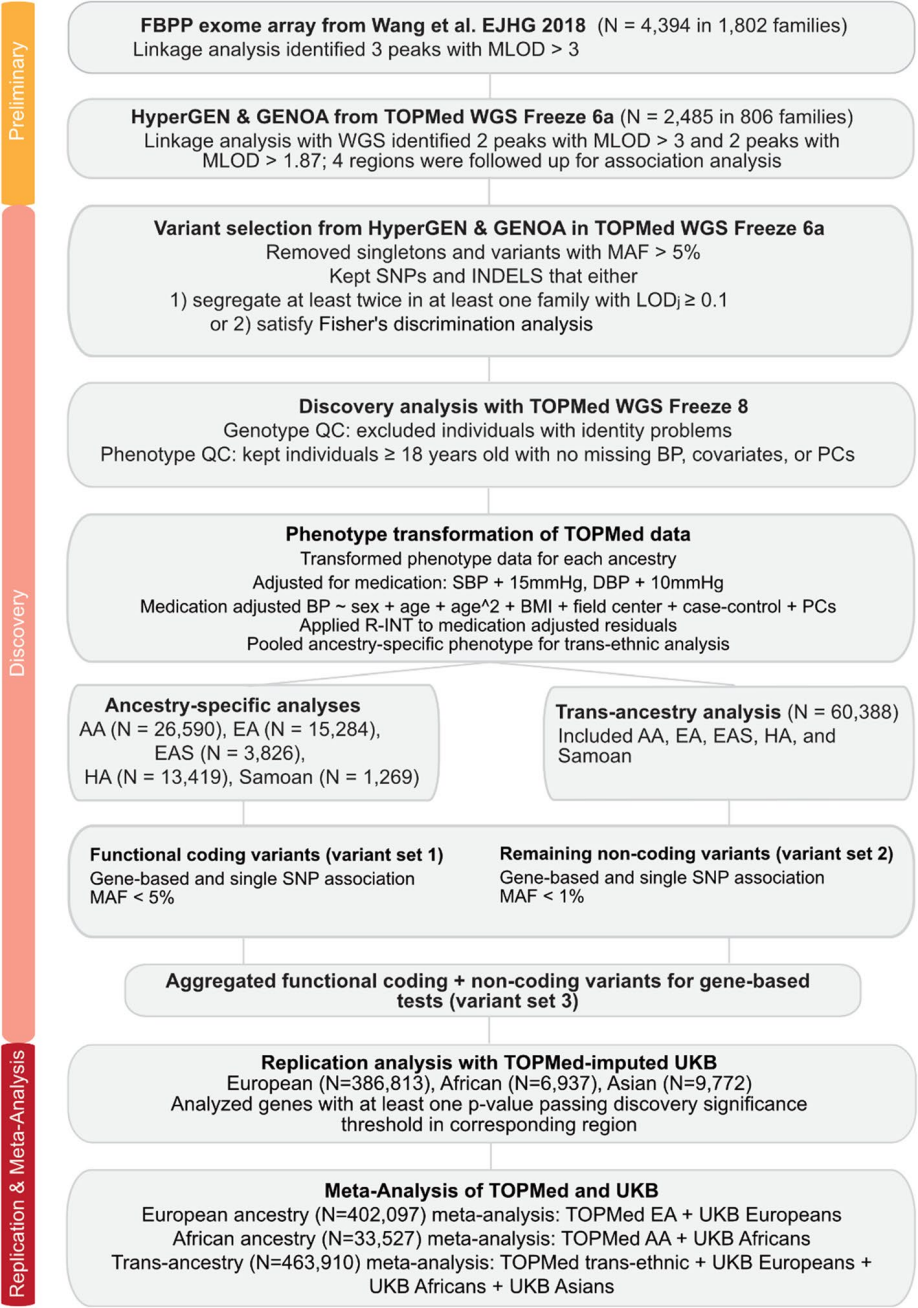
Overview of analysis workflow. Abbreviations: MLOD (maximum LOD score), LOD_j_ (family-specific LOD score for family *j*), QC (quality control), PCs (principal components), R-INT (rescaled inverse normal transformation), AA (African American), EA (European American), EAS (Eastern Asian/Asian American), HA (Hispanic American)

**Fig. 2:**
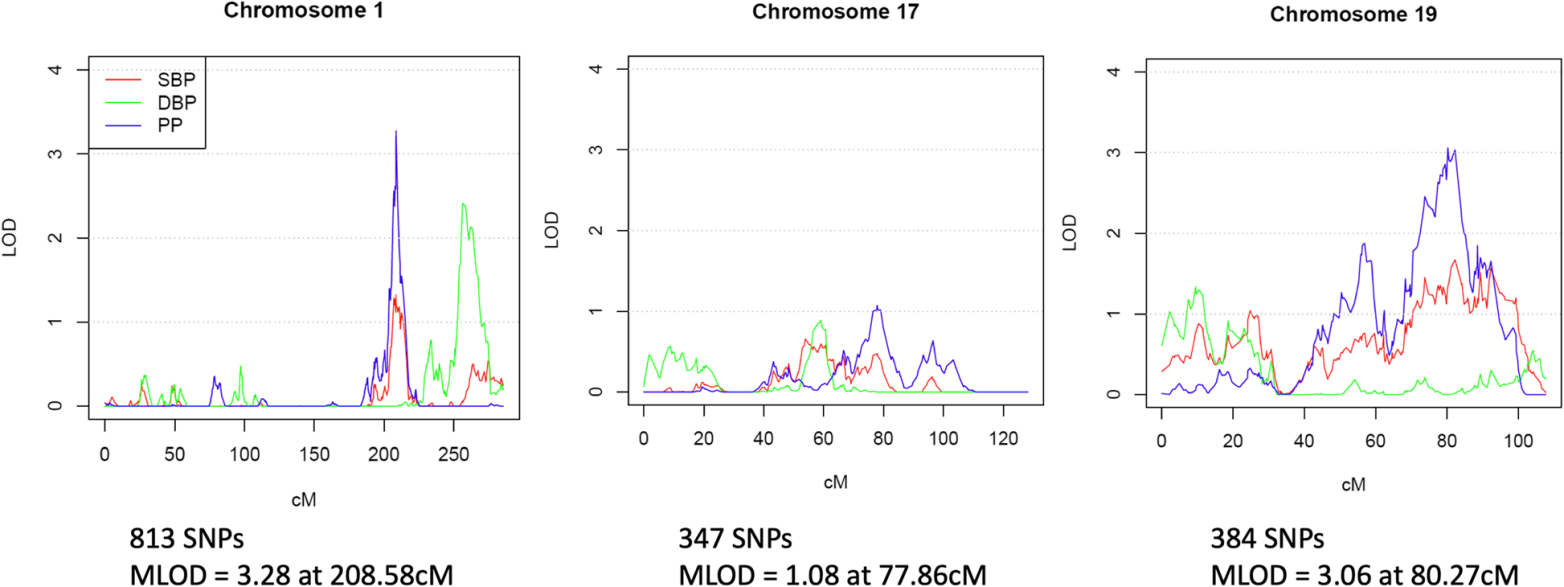
Linkage analysis with HyperGEN and GENOA subjects in TOPMed Freeze 6a release. Abbreviations: SBP (systolic blood pressure); DBP (diastolic blood pressure); PP (pulse pressure); cM (centimorgan); LOD (logarithm of the odds); MLOD (maximum LOD score)

The overall analysis workflow is illustrated in Fig. 1. After conducting linkage analysis on chromosomes 1, 17, and 19 using TOPMed Freeze 6a WGS data, the linkage peaks on chromosomes 1 and 19 from Wang et al. [11] remained but the peak on chromosome 17 was no longer significant (Fig. 2). There were 2 significant linkage peaks for PP on chromosome 1q31 (maximum LOD = 3.28) and chromosome 19q13.33 (MLOD = 3.06). Two additional regions with maximum LOD > 1.87 were followed up on chromosomes 1 and 19: 1q42 for DBP (maximum LOD = 2.41) and 19q13.11 for PP (maximum LOD = 1.87). These four genomic regions on chromosomes 1 and 19 were followed-up for association analysis.

### Discovery association analyses with TOPMed WGS data

Discovery gene-based association analyses were completed for variant set 1, 2, and 3 in each of the four linkage regions and a Bonferroni correction adjusting for the number of genes tested was applied in each region to establish four discovery significance thresholds. Any genes with at least one *p*-value in any trait or any variant group passing the corresponding region’s discovery significance threshold were followed up with additional analyses (Table 1). There were four genes from 1q31, seven genes from 1q42, four genes from 19q13.11, and 12 genes from 19q13.33 that passed the corresponding thresholds. Of these 18 genes, variant set 1 (low frequency coding variants) of *RCN3*, reticulocalbin 3, showed the strongest association evidence for DBP in TOPMed trans-ancestry samples (burden *p* = 1.36 × 10^− 5^; beta = − 0.051, Tables 2 and 3). One variant (rs146159696) overlapped between coding and non-coding variant sets as it is both a missense and intronic variant for different transcripts. This variant also has the most significant *p*-value in the single variant association analysis (DBP *p* = 1 × 10^− 4^). The association direction of these coding variants in EA, AA and HA ancestries were consistent and neither EAS nor Samoan cohorts carried these variants. These 18 genes were carried forward for replication analysis in UKB and gene expression association analysis with GTEx.

**Table 1:** Genes passing discovery significance threshold in each linkage region

	1q31 (52 genes; *p* < 9.62 × 10^− 4^)	1q42 (29 genes; *p* < 1.72 × 10^− 3^)	19q13.11 (112 genes; *p* < 4.46 × 10^− 4^)	19q13.33 (354 genes; *p* < 1.41 × 10^− 4^)
AA	*RGS18* *RGS13* *LAD1*		*CCNE1*	*PLEKHA4* *CD37*
EA	*CACNA1S*	*SLC35F3* *COA6*		*SNRNP70* *SIGLECL1*
EAS			*WDR88*	
HA			*ARHGAP33*	*ZNF665*
Samoan				*VSIG10L*
Trans-ancestry		*RYR2*		*RCN3* *GFY*

*Abbreviations: AA* African Americans, *EA* European Americans, *EAS* East Asians/Asian Americans, *HA* Hispanic Americans

**Table 2:** Gene-based analysis summary statistics of *RCN3* in TOPMed Freeze 8 and UK Biobank: coding variants with MAF < 5%

		PP			SBP			DBP		
	NVAR	Beta	Burden P	SKAT P	Beta	Burden P	SKAT P	Beta	Burden P	SKAT P
**TOPMed (discovery)**										
**AA (*N*** **= 26,590)**	7	0.027	0.351	0.543	−0.010	0.752	0.885	−0.057	5.75 × 10^−3^	0.092
**EA (*N*** **= 15,284)**	3	0.008	0.694	0.833	−0.033	0.228	0.411	−0.082	6.85 × 10^−4^	4.34 × 10^−3^
**EAS (*N*** **= 3826)**	0	NA	NA	NA	NA	NA	NA	NA	NA	NA
**HA (*N*** **= 13,419)**	5	−0.018	0.571	0.580	−0.051	0.240	0.135	−0.043	0.099	0.035
** Samoan (*N*** **= 1269)**	0	NA	NA	NA	NA	NA	NA	NA	NA	NA
** Trans-ancestry (*N* = 60,388)**	7	0.011	0.411	0.805	−0.022	0.178	0.218	− 0.051	1.36 × 10^−5^	8.11 × 10^−5^
**UKB (replication)**										
**African (*N*** **= 6937)**	4	−0.124	0.004	0.007	−0.249	**5.90 × 10**^**− 5**^	**4.43 × 10**^**− 4**^	− 0.126	**3.93 × 10**^**− 4**^	0.005
**European (*N*** **= 386,813)**	4	0.021	0.004	0.006	0.034	**5.80 × 10**^**− 4**^	**6.15 × 10**^**− 4**^	0.013	0.018	0.011
**Asian (*N*** **= 9772)**	3	−0.087	0.391	0.305	−0.095	0.503	0.514	−0.009	0.906	0.966

*Abbreviations*: *NVAR* Number of linkage-based selected variants passing all filters for analysis), *SBP* Systolic blood pressure, *DBP* Diastolic blood pressure, *PP* Pulse pressure, *AA* African Americans, *EA* European Americans, *EAS* East Asians/Asian Americans, *HA* Hispanic Americans, *SKAT* Sequence Kernel Association Test

Bolded *p*-values in UKB indicate significance after Bonferroni correction for 18 genes

**Table 3:** Gene-based analysis summary statistics of *RCN3* in TOPMed Freeze 8 and UK Biobank: non-coding variants with MAF < 1%

		PP			SBP			DBP		
	NVAR	Beta	Burden P	SKAT P	Beta	Burden P	SKAT P	Beta	Burden P	SKAT P
**TOPMed (discovery)**										
**AA (*N* = 26,590)**	267	7.52 × 10^− 4^	0.774	0.262	0.003	0.268	0.837	−0.002	0.314	0.073
**EA (*N* = 15,284)**	130	0.003	0.288	0.949	−1.68 × 10^−4^	0.967	0.969	0.004	0.299	0.496
**EAS (*N* = 3826)**	51	0.020	0.294	0.469	0.021	0.386	0.279	4.38 × 10^−4^	0.980	0.323
**HA (*N* = 13,419)**	189	−5.61 × 10^−4^	0.891	0.714	0.002	0.704	0.929	0.002	0.553	0.751
**Samoan (*N* = 1269)**	30	0.063	0.318	0.148	0.200	0.049	0.090	0.123	0.079	0.296
**Trans-ancestry (*N* = 60,388)**	267	0.002	0.200	0.902	2.72 × 10^−4^	0.880	0.984	−2.56 × 10^− 4^	0.843	0.183
**UKB (replication)**										
** African (*N* = 6937)**	184	0.005	0.355	0.309	0.004	0.630	0.815	−1.31 × 10^−3^	0.762	0.488
** European (*N* = 386,813)**	145	−1.03 × 10^− 3^	0.407	**2.97 × 10**^**−4**^	−0.002	0.361	**4.67 × 10**^**−5**^	−7.97 × 10^− 4^	0.387	0.017
**Asian (*N* = 9772)**	65	0.014	0.029	0.024	0.015	0.088	0.126	0.001	0.868	0.889

Bolded *p*-values in UKB indicate significance after Bonferroni correction for 18 genes

*Abbreviations: NVAR* number of linkage-based selected variants passing all filters for analysis, *SBP* systolic blood pressure, *DBP* diastolic blood pressure, *PP* pulse pressure, *AA* African Americans, *EA* European Americans, *EAS* East Asians/Asian Americans, *HA* Hispanic Americans, *SKAT* Sequence Kernel Association Test

### Replication association analyses of unrelated samples in TOPMed-imputed UK Biobank

Independent replication analysis was performed using the UKB TOPMed-imputed genotype data and baseline phenotype data. The two variant sets described in the Methods section were analyzed using GENESIS [13]. The top gene from the UKB replication analysis was also *RCN3*. Coding variants of *RCN3* were nominally associated with all three BP traits in the two gene-based association tests for Europeans and Africans, with the lowest *p*-value being burden *p* = 5.90 × 10^− 5^ for SBP, which also significant after Bonferroni correction for multiple comparisons (18 genes × 2 independent traits × 3 variant sets × 3 ethnic populations × 2 statistical tests).

### Meta-analyses of TOPMed and UK Biobank

Finally, trans-ancestry meta-analysis and ancestry-specific meta-analyses for European and African ancestries were conducted for *RCN3* in all variant sets (Tables 4) using TOPMed and UKB data. In the trans-ancestry meta-analysis, gene-based association test of variant set 1 (low frequency coding variants) in *RCN3* reached genome-wide significance for DBP (burden *p* = 2.01 × 10^− 7^), which was also significant after adjusting for multiple testing (547 genes from Table 1 × 2 independent traits × 3 variant sets × 2 statistical tests). Among all individuals of European ancestry, *RCN3* variant set 1 was also significant (burden *p* = 3.88 × 10^− 6^) with DBP after adjusting for multiple tests (547 genes × 2 independent traits × 3 variant sets × 2 statistical tests). Among all individuals of African ancestry, we also observed suggestive evidence for DBP (burden *p* = 3.16 × 10^− 5^). Finally, when coding and noncoding variants are combined (set 3), the association evidence of *RCN3* gene remained, although the *p*-values were slightly inflated (Table 4). The variants in this set were further examined in single SNP association analysis (Table 5). Ancestry-specific single SNP association results are shown for SNPs that were observed in both TOPMed and UKB. For *RCN3*, there were seven low frequency and rare coding variants selected using linkage evidence in African-American families in HyperGEN and GENOA. Of those seven variants, three can be found in TOPMed EA (rs142564622, rs34218348, and rs146159696), all of which were also observed in the UKB European data plus an additional variant (rs770319784).

**Table 4:** Meta-Analysis *p*-values of *RCN3* in TOPMed Freeze 8 and UK Biobank

		PP		SBP		DBP	
	DF	Burden	SKAT	Burden	SKAT	Burden	SKAT
**Variant set 1 (coding variants with MAF < 5%)**							
**TOPMed EA + UKB European**	4	0.012	0.032	0.001	0.001	**3.88 × 10**^**−6**^	1.29 × 10^−5^
**TOPMed AA + UKB African**	4	0.010	0.024	4.89 × 10^−4^	0.003	3.16 × 10^−5^	0.004
**TOPMed + UKB**	8	1.15 × 10^−3^	0.003	**4.49 × 10**^**−6**^	3.15 × 10^− 5^	**2.01 × 10**^**− 7**^	**5.43 × 10**^**− 6**^
**Variant set 2 (noncoding variants with MAF < 1%)**							
**TOPMed EA + UKB European**	4	0.285	0.002	0.682	5.04 × 10^− 4^	0.691	0.022
**TOPMed AA + UKB African**	4	0.630	0.284	0.469	0.943	0.581	0.154
**TOPMed + UKB**	8	0.077	9.49 × 10^−4^	0.425	0.002	0.930	0.106
**Variant set 3 (coding variants with MAF < 5% + noncoding variants with MAF < 1%)**							
**TOPMed EA + UKB European**	4	0.267	0.002	0.692	5.08 × 10^− 4^	0.614	0.019
**TOPMed AA + UKB African**	4	0.771	0.136	0.621	0.510	0.357	0.063
**TOPMed + UKB**	8	0.136	1.12 × 10^−3^	0.644	3.91 × 10^−4^	0.845	0.017

*Abbreviations: DF* degrees of freedom, *SBP* systolic blood pressure, *DBP* diastolic blood pressure, *PP* pulse pressure, *AA* African Americans, *EA* European Americans, SKAT Sequence Kernel Association Test

Meta-analysis of TOPMed and UKB were calculated using Fisher’s method with 2 k degrees of freedom, where k = 4 (one TOPMed trans-ancestry analysis and three UKB ancestry-specific analyses for individuals of European, African, and Asian ancestries)

Bolded *p*-values represent significance after Bonferroni correction for multiple comparisons (547 genes × 2 independent traits × 3 variant sets × 2 statistical tests)

**Table 5:** Single variant association tests of *RCN3* coding variants in TOPMed Freeze 8 and UK Biobank

					PP			SBP			DBP		
Variant	Effect	Ancestry	N	Frequency	Beta	SE	*P*-value	Beta	SE	*P*-value	Beta	SE	*P*-value
19:49534206_C/A (rs1004941866)	Missense	TOPMed trans-ancestry	60,387	2.484 × 10^−5^	14.01	10.36	0.18	7.28	14.44	0.61	−4.12	7.44	0.58
19:49537166_C/A (rs142564622)	Missense	TOPMed trans-ancestry	60,384	7.204 × 10^−4^	2.13	1.35	0.11	−1.29	1.77	0.47	−2.94	1.33	0.03
		TOPMed EA	26,587	1.034 × 10^−3^	0.98	1.58	0.54	−2.79	2.08	0.18	−4.51	1.81	0.01
		TOPMed AA	15,283	5.23 × 10^−4^	5.29	3.80	0.16	−0.72	4.82	0.88	−4.71	2.76	0.09
		UKB European	386,813	1.72 × 10^−4^	1.61	1.18	0.17	1.07	1.61	0.51	−0.58	0.87	0.51
		UKB African	6937	5.77 × 10^−4^	−3.77	4.58	0.41	−3.83	3.78	0.31	−8.02	6.61	0.23
19:49539182_G/T (rs374733821)	Splice region	TOPMed trans-ancestry	60,385	1.076 × 10^−4^	−6.54	4.42	0.14	−4.58	5.26	0.38	0.31	2.71	0.91
19:49542553_C/T (rs376990460)	Missense	TOPMed	60,387	7.452 × 10^−5^	− 4.64	5.92	0.43	−2.08	6.97	0.77	1.78	3.55	0.62
19:49542684_C/G (rs34218348)	Missense	TOPMed trans-ancestry	60,386	5.539 × 10^−3^	0.25	0.52	0.64	−0.01	0.57	0.99	−0.64	0.40	0.11
		TOPMed EA	26,590	3.761 × 10^−5^	−22.02	12.61	0.08	−17.23	15.85	0.28	2.91	12.49	0.82
		TOPMed AA	15,283	0.02	0.14	0.60	0.81	−5.02	5.16	0.33	−3.45	2.80	0.22
		UKB European	193,427	3.36 × 10^−5^	−1.90	3.77	0.61	−5.05	5.16	0.33	−3.46	2.80	0.22
		UKB African	6937	0.016	−2.42	0.87	0.01	−1.97	0.72	0.01	−4.39	1.26	4.99 × 10^− 4^
19:49542722_C/A (rs146159696)	Missense	TOPMed trans-ancestry	60,386	0.010	0.05	0.34	0.88	−0.72	0.43	0.10	−1.28	0.323	1.00 × 10^−4^
		TOPMed EA	26,590	0.013	0.11	0.41	0.78	−0.40	0.52	0.44	−1.28	0.47	6.04 × 10^−3^
		TOPMed AA	15,282	3.50 × 10^− 3^	0.20	1.41	0.88	−1.16	1.56	0.45	−1.62	0.92	0.08
		UKB European	386,813	0.02	0.34	0.12	0.01	0.59	0.17	5.71 × 10^−4^	0.24	0.09	0.01
		UKB African	6937	1.08 × 10^−3^	−0.20	3.36	0.95	−1.60	2.77	0.57	−1.92	4.84	0.69
19:49543160_AG/A (rs770319784)	Frameshift	TOPMed trans-ancestry	60,385	3.643 × 10^−4^	2.85	2.42	0.24	1.93	2.84	0.50	−1.35	1.64	0.41
		TOPMed AA	15,283	1.28 × 10^−3^	1.93	2.61	0.46	0.72	2.99	0.81	−2.11	1.75	0.23
		UKB European	38,689	2.58 × 10^−5^	−10.44	9.62	0.28	−10.52	13.14	0.42	−2.37	7.137	0.74
		UKB African	6937	1.80 × 10^−3^	−2.37	2.60	0.36	−4.56	2.14	0.03	−6.63	3.74	0.08

*Abbreviations: N* number of samples analyzed, *AA* African Americans, *EA* European Americans, *SKAT* Sequence Kernel Association Test

### Gene expression association analysis

**Fig. 3:**
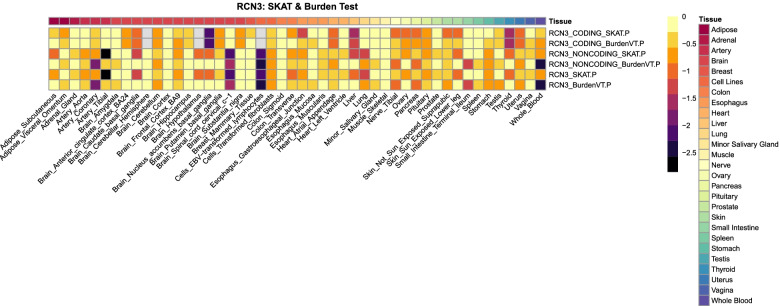
Heat map of *p*-values from GTEx tissue-specific gene expression association analysis. Gene expression-association analyses were conducted in EPACTS using variable threshold burden test (BurdenVT) and Sequence Kernel Association Test (SKAT)

Tissue-specific gene expression association analyses were completed for 18 genes of interest using GTEx v7 WGS data (*N* = 635) and *cis*-eQTL gene expression data in 48 tissues (including 2 cell lines). The availability of gene expression data varies by tissue along with varying sample size on a tissue-by-tissue basis. For *RCN3*, gene expression gene-based tests were completed for coding variants only, noncoding variants only, and the aggregated set. *P*-values from SKAT and burden tests are illustrated on a heat map (Fig. 3). Although none of the associations passed the Bonferroni correction (*p* = 0.05/(48×2) = 5.2 × 10^− 4^), the heat map shows that *RCN3* variants were nominally associated with gene expression in multiple tissues of the artery, brain, and thyroid, which have shown to be relevant to BP regulation [14, 15].

## Discussion

This study showed that leveraging linkage evidence from family-based studies could effectively and efficiently detect rare variants associated with complex BP traits. This approach successfully identified rare variants associated with BP traits without conducting computationally intensive sliding window-based association analysis across the whole genome and running a large number of tests. Therefore, our approach can be considered as complementary to genome-wide based approaches, which may miss the rare variants or genes identified in this study. Though the variants included for analysis were initially selected from AA families, association evidence for the genes can be observed and replicated well in independent multi-ancestry samples, including African ancestry samples (Tables 1 and 4), demonstrating the robustness of using linkage evidence to guide association analysis of low frequency and rare variants. Across multiple ancestries, we observed evidence of allelic heterogeneity as the top genes in ancestry-specific analyses included low frequency and rare variants that are more common or specific to their corresponding ancestries.

Meanwhile, it is also challenging to study rare variants in trans-ancestry samples as many rare variants are ancestry-specific due to their rarity. Because the sample sizes for non-European cohorts are often much smaller, the statistical power is reduced and replication may be challenging. For example, low frequency coding variants of *VSIG10L* demonstrated suggestive association evidence for DBP in the Samoan Adiposity Study (burden *p* = 9.24 × 10^− 6^; beta = 0.521), but not in any other ancestry. The significant gene-based test was mostly driven by a single variant rs141732375 (*p* = 9.82 × 10^− 5^; beta = 7.01). Due to data availability, replication in other Samoan cohorts was not feasible at the time of the analysis.

The significant gene after correcting for multiple testing identified from this study was *RCN3*. The association of the *RCN3* coding variants in samples of African ancestry has *p*-values of 0.01, 4.89 × 10^− 4^ and 3.16 × 10^− 5^ for PP, SBP and DBP, respectively (Table 4), despite the relatively small sample size. This association evidence is consistent with the linkage evidence. Similar association is also present in samples of European ancestry with larger sample size. It is encouraging to observe that rare coding variants in *RCN3* are associated with both AA and EA in UKB replication analysis (Table 2), suggesting the association evidence is not a false positive.

However, the association evidence for non-coding variants (variant set 2) was less consistent because TOPMed cohorts did not show any association evidence for BP traits, but *RCN3* non-coding variants in UKB European samples showed significant association evidence in SKAT for PP (*p* = 2.97 × 10^− 4^) and SBP (*p* = 4.67 × 10–5) after adjusting for multiple comparisons (Table 3). In the single SNP association analysis (Table 5), there were seven low frequency or rare coding variants identified from HyperGEN and GENOA using the approach described in the Methods section. Among European ancestry samples, three out of seven SNPs (rs142564622, rs34218348, and rs146159696) were observed in both TOPMed EA and UKB European, and one SNP (rs770319784) was only observed in UKB European. The four SNPs observed in UKB European were also observed in UKB African. For the four SNPs observed in both TOPMed and UKB, the directions of effect in DBP was completely consistent for rs142564622 and rs770319784 and nearly consistent for rs34218348 and rs146159696.

The *p*-values in the discovery stage might be inflated because linkage analysis and variant selection for association analysis were performed within the same pedigrees. Our previous simulation study suggested such inflation is minimal [8]. However, to be conservative, we used the Bonferroni-corrected *p*-value threshold in the UKB replication data (*p* = 7.72 × 10^− 5^) after correcting for 2 independent BP traits, 2 statistical tests, 3 variant sets, and 3 UKB populations (European, African, Asian), and 18 genes. For the trans-ethnic TOPMed and UKB meta-analysis, a p-value threshold of 7.62 × 10^− 6^ was used to declare significance after adjusting for 547 genes, 2 independent traits, 3 variant sets and 2 statistical methods. Thus, the association evidence of *RCN3* with DBP and SBP reported in this study is significant in both UKB replication data as well as combined TOPMed and UKB trans-ethnic data.

There is some genetic evidence supporting the association between *RCN3* and BP traits. UKB GWAS by Neale et al. [16] found two genome-wide significant SNPs associated with hypertension: rs61760904 (missense; non-Finnish European allele frequency = 0.007; OR = 1.2; p-value = 1.8E × 10^− 9^; CADD > 23.4) and rs73046792 (3′ UTR variant; non-Finnish European allele frequency = 0.15; OR = 0.96; p-value = 3.6 × 10^− 8^; CADD > 0.89). One SNP downstream of *RCN3*, rs189349094, is associated with systolic blood pressure [17] and linked to *RCN3* through GeneHancer [18]. None of these previously reported SNPs overlap with SNPs selected by linkage evidence, suggesting the variants we identified in *RCN3* are novel.

One pattern observed in the gene-based association analysis was that the strongest association evidence did not come from PP, the trait with the linkage signal. One possible explanation is that when the directions of effect are the same for SBP and DBP, the effect size for PP is reduced because PP is the difference of SBP and DBP; thus, canceling the association of PP.

There are a number of known imputation challenges for rare variants, particularly for non-European individuals, in the UKB data imputed using the Haplotype Reference Consortium [19]. Therefore, it was necessary to re-impute these regions using the multi-ancestry TOPMed reference panel. Unpublished results from our group and recent TOPMed publications have shown that the TOPMed reference panel can successfully impute rare variants found in populations of African ancestry [12, 20, 21]. With the TOPMed imputation, we were able to examine UKB samples with European, African, and Asian ancestries.

Common genetic variants discovered from GWAS face a challenge of pinpointing causal genes and therefore are difficult to interpret. On the other hand, rare variants may contribute to a trait’s “missing heritability” but are extremely difficult to uncover and to replicate due to insufficient statistical power for currently available samples with WGS data, such as TOPMed. The primary goal of this study is to search for rare variants using the TOPMed WGS data with an approach that is not widely used in WGS association analysis. However, our study demonstrates that this approach can be successful in identifying rare variants and is complementary to purely population-based approaches. The association of the coding variants identified in *RCN3* gene is replicable and present across multiple ancestries, although the original linkage evidence was identified from AA families. Additionally, these coding variants are more interpretable; however, further functional studies are needed to understand the mechanisms underlying how these variants contribute to BP variation.

There are some limitations of our study. The major limitation is the wide range of study designs and phenotype collection procedures in the studies included. While adjustments were included in analyses for study and data collection centers, it was difficult to control for the study design differences, which may reduce statistical power.

## Conclusions

This study examined low frequency and rare variants under linkage peaks on chromosomes 1 and 19 that were detected in AA families. By focusing on linkage regions and following up with gene-based and single SNP association analyses, multiple genes were found to be associated with BP traits. In particular, low frequency and rare coding variants from *RCN3* were significantly associated with DBP in trans-ancestry samples. While our finding is supported by genetic evidence, additional analyses are warranted to examine the underlying biological mechanisms. This study demonstrates that leveraging linkage evidence in WGS expedites the process of identifying functional rare variants associated with complex traits. Individually, these rare variants might only explain a small portion of heritability in the population level, but they could facilitate our understanding of the genetic determinants of hypertension in diverse populations. Additionally, functional rare variants identified from this type of study could further facilitate the identification of disease targets.

## Methods

### Study population

The discovery analysis included all TOPMed Freeze 8 samples with the harmonized BP phenotype at the time of analysis, which consisted of 18 TOPMed studies (32 ancestry- and study-specific cohorts). These 18 studies (*N* = 60,388) included 26,590 EA, 15,284 AA, 3826 East Asians or Asian Americans (EAS), 13,419 Hispanic Americans (HA), and 1269 Samoans from the following studies: Genetics of Cardiometabolic Health in the Amish (Amish; EA), Atherosclerosis Risk in Communities Study from the Venous Thromboembolism (VTE) project (ARIC; EA and AA), Mount Sinai BioMe Biobank (BioMe; EA, AA, EAS, and HA), Coronary Artery Risk Development in Young Adults (CARDIA; EA and AA), Cleveland Family Study (CFS; EA and AA), Cardiovascular Health Study (CHS; EA and AA), Framingham Heart Study (FHS; EA), Genetic Epidemiology Network of Salt Sensitivity (GenSalt; EAS), Genetic Studies of Atherosclerosis Risk (GeneSTAR; EA and AA), Hispanic Community Health Study – Study of Latinos (HCHS_SOL; HA), Hypertension Genetic Epidemiology Network and Genetic Epidemiology Network of Arteriopathy (HyperGEN_GENOA; AA), GENOA from the African American Coronary Artery Calcification project (part of HyperGEN_GENOA; AA), Jackson Heart Study (JHS; AA), Multi-Ethnic Study of Atherosclerosis (MESA; EA, AA, EAS, and HA), MESA Family Study from the African American Coronary Artery Calcification project (MESAFam; AA), San Antonio Family Studies (SAFS; HA), Samoan Adiposity Study (Samoan), Taiwanese Study of Hypertension using Rare Variants (THRV; EAS), and the Women’s Health Initiative (WHI; EA, AA, EAS, and HA). These studies vary in design: BioMe, CARDIA, CHS, HCHS_SOL, and the Samoan study are primarily community-based studies; JHS and MESA are community-based studies that include a nested family-based design; Amish, CFS, FHS, GeneSTAR, GenSalt, HyperGEN_GENOA, MESAFam, SAFS, and THRV are family-based studies; and ARIC and WHI are population-based cohort studies in which case-control samples were selected for TOPMed. Descriptions of these studies and data collection procedures are included in (Additional File 1. Supplemental Materials & Methods).

The UK Biobank (UKB) version 3 GWAS data [22] were used as the replication cohort. These samples were collected from across the United Kingdom from participants between 40 to 69 years old. The UKB replication cohort included individuals of European ancestry (*N* = 417,634), African ancestry (*N* = 7297), and Asian ancestry (*N* = 10,215). Ethnic subgroups were clustered. Individuals with ethnic subgroup coding of 1 (White), 1001 (British), 1002 (Irish), and 1003 (any other white background) were considered as European ancestry, those with coding of 4 (Black or Black British), 4001 (Caribbean), 4002 (African), 4003 (any other black background) were considered as African ancestry, and those with coding of 3 (Asian), 3001 (Indian), 3002 (Pakistani), 3003 (Bangladeshi), 3004 (any other Asian background), and 5 (Chinese) were considered as Asian ancestry.

### Genotyping and quality control (QC)

The TOPMed Informatics Research Center (IRC) and Data Coordinating Center (DCC) centrally performed sample and genotype quality control (QC). Detailed QC procedures are described in the TOPMed flagship paper [12] and TOPMed Freeze 8 website (https://topmed.nhlbi.nih.gov/topmed-whole-genome-sequencing-methods-freeze-8). The software BCFtools [23] was used to apply the following QC filters: 1) bi-allelic single nucleotide polymorphisms (SNPs) and small insertion-deletion polymorphisms (INDELs) passing all genotype filters; 2) a minimum 10x sequencing depth. The participant must not have any known identity problems (such as sex or pedigree mismatches) reported by the DCC to be included for analysis. In this study, unique participants from 18 TOPMed studies from the Freeze 8 release (GRCh38) were included, reflecting the May 30, 2019 sample annotation from the TOPMed DCC. After excluding individuals under 18 years old and those with missing BP measurements or covariates, the combined study sample contained 60,388 individuals. Principal components (PCs) and kinship matrix were both made available by the TOPMed DCC. As described in the TOPMed Flagship paper [12], the PCs were calculated using PC-AiR [13], and the kinship matrix was calculated using the *pcrelate* function in the GENESIS R package [24]. This approach estimates kinship coefficients and identical-by-descent (IBD) sharing probabilities conditional on ancestry. A fourth-degree sparse kinship matrix provided by TOPMed was used as the covariance matrix in the linear mixed model for optimal computational efficiency. The TOPMed DCC has determined that the top 11 PCs well represent global ancestry patterns among TOPMed Freeze 8 samples. Therefore, these PCs were adjusted in the phenotype residuals and linear mixed model to account for genetic ancestry background.

The UKB data were genotyped using the Affymetrix UK Biobank Axiom array [22]. Principal components were calculated by UKB with genotype data within each ancestry to account for population structure (http://www.ukbiobank.ac.uk/wp-content/uploads/2014/04/UKBiobank_genotyping_QC_documentation-web.pdf). Because the UKB imputed genotype data were originally imputed using the Haplotype Reference Consortium [25] reference panel, which is predominantly of European ancestry, we re-imputed Europeans, Africans, and Asians using the TOPMed reference panel. The TOPMed reference panel is a diverse reference panel including information from 97,256 deeply sequenced human genomes, and we were able to impute rare variants for non-European individuals with high confidence (r^2^ > 0.3). Ancestry-specific genotype imputation was conducted on the TOPMed Imputation Server (https://imputation.biodatacatalyst.nhlbi.nih.gov/). The software QCTOOL v2 (https://www.well.ox.ac.uk/~gav/qctool_v2/index.html) was used to convert the BGEN format genotype files to VCF format. The following pre-imputation quality control were done in PLINK 1.9 [26]: variants with MAF < 1%, genotyping rate < 97%, or Hardy-Weinberg Equilibrium < 1 × 10^− 6^ were removed. Variants were remapped from GRCh37 to GRCh38 using the TOPMed Imputation Server, and those variants that cannot be remapped were excluded. Imputed variants with a r^2^ > 0.3 were retained for analysis. Further analysis by increasing the threshold to r^2^ > 0.5 did not affect the result (Additional file 1. Supplemental Materials and Methods). Sample QC was performed for UKB by excluding outliers in heterozygosity and missing rates defined by UKB.

The SeqArray R package [27] was used to convert VCF format into GDS format to be used in the GENESIS R package [13] for association analysis. Related individuals with pairwise kinship coefficient greater than 0.0884 [28], which is the threshold for third degree relatives calculated using software KING [29], were removed from analysis, resulting in 386,813 individuals of European ancestry, 6937 individuals of African ancestry, and 9772 individuals of Asian ancestry from the UKB.

### Phenotype harmonization

TOPMed phenotype data were collectively harmonized by members of the TOPMed BP Working Group. Details on TOPMed phenotype harmonization for systolic blood pressure (SBP), diastolic blood pressure (DBP), and pulse pressure (PP) were described in our previous study [7]. Covariates used in the analyses were measured at the same visit as the BP measurements.

For the UKB cohort, baseline BP and covariates (Additional file 2. Table S1) were extracted from the phenotype data. Because two SBP and DBP measurements were taken at baseline, the average of the two measurements was used to generate the phenotypes for association analyses. Individuals with missing BP data at baseline were excluded from analysis.

### Transformation of phenotype data for association analyses

As each TOPMed project has a different study design and sample population, it is important to standardize the quantitative trait values by applying data transformation and rescale to restore the original measurement for genetic effects. In this study, the phenotype residuals were calculated separately by ancestry and phenotype transformation was applied to account for between-study heterogeneity. Harmonized BP phenotypes were pooled within each ancestry and BP traits were adjusted for anti-hypertensive medications use by adding 15 mmHg and 10 mmHg to raw SBP and DBP measurements, respectively [30]. The regression residuals were calculated for medication-adjusted SBP, DBP, and PP after adjusting for age, age^2^, sex, body mass index (BMI), field center (for multi-center studies), case-control status for stroke or venous thromboembolism (WHI only), and the top 11 PCs. Next, inverse normal transformation was applied to the ancestry-specific residuals. The inverse normal transformed residuals were re-scaled using the standard deviation (SD) of raw BP measurement, prior to medication adjustment, in each study. This results in a rescaled inverse normal transformation (R-INT) that makes the phenotype to follow a normal distribution and restores the original scale of measurement [31]. The phenotype distributions and transformations are shown in (Additional files 3, 4, 5, 6 and 7: Figs. S1-S5).

The R-INT residuals of BP phenotypes were analyzed in both gene-based and single variant association analyses. The covariates described above were adjusted for the second time in the linear mixed model. Previously, Softer et al. used TOPMed data to show that a two-stage approach to adjust for covariates can improve statistical power and reduce type I error [32]. Ancestry-specific phenotypes were pooled for the trans-ethnic analysis in TOPMed. In the UK Biobank data analysis, SBP and DBP were adjusted for anti-hypertensive medications use by adding 15 mmHg and 10 mmHg, respectively. Covariates (age, BMI, assessment center) and top 10 PCs were included in the same way as described for TOPMed data.

### Overview of statistical methods

The overall analysis workflow includes 3 stages and is illustrated in Fig. 1. In the preliminary stage, we conducted linkage analysis with AA families in HyperGEN and GENOA using TOPMed WGS data. In the discovery stage, we completed gene-based and single variant association analyses using the SNPs prioritized by linkage evidence. In the final stage, we performed replication for the top genes identified from the discovery stage in the TOPMed-imputed UK Biobank data and meta-analyzed TOPMed with UK Biobank by ancestry and across ancestries.

### Linkage analysis of AA families with TOPMed WGS data

We performed multi-point variance-component linkage analysis of TOPMed WGS data in HyperGEN and GENOA families to obtain the family-specific LOD scores. Study-specific BP residuals, after adjusting for anti-hypertensive medication use, were used in the linkage analysis. The genetic map for GRCh38 was obtained from the University of Washington (http://bochet.gcc.biostat.washington.edu/beagle/genetic_maps/). The set of linkage disequilibrium pruned SNPs that was used in the exome array linkage analysis by Wang et al. [11] (MAF > 0.2 and linkage disequilibrium r^2^ < 0.1), which consists of 813 markers for chr1, 347 markers for chr17, and 384 markers for chr19, was used again in the linkage analysis with TOPMed WGS data. Linkage region was defined as a two-LOD score drop from the linkage peak SNP, which has the highest LOD score.

The linkage regions were re-defined using WGS data due to two key reasons: 1) only 3085 out of 4394 individuals (70%) could be found in both FBPP exome array data analyzed by Wang et al. [11] and TOPMed WGS data; 2) there were several pedigree relatedness problems with the exome array data (e.g. half/step siblings were separated into different families), which resulted in inaccurate family-specific LOD scores. After correcting the pedigree errors, multi-point variance-component linkage analysis was conducted using MERLIN [33] for three BP traits (SBP, DBP, and PP) on chromosomes 1, 17, and 19 using 3149 HyperGEN and GENOA individuals in the TOPMed Freeze 6a release, the latest release at the time of analysis. For HyperGEN and GENOA, the individuals are identical for Freeze 6a and Freeze 8. Chromosome 17 was excluded from further analysis due to a lack of linkage evidence.

### Variant selection from HyperGEN & GENOA families in TOPMed WGS

In the preliminary stage, we performed variance component linkage analysis in African-American families and searched for linkage regions with suggestive linkage evidence. Single SNP and gene-based associations for selected variants were conducted in protein-coding genes within the linkage regions on 1q31 (chr1:188765880–202,026,147), 1q42 (chr1:232963435–240,632,149), 19q13.11 (chr19:22332449–36,438,656), and 19q13.33 (chr19:41978814–53,404,335). We examined two significant linkage peaks (maximum LOD > 3) on 1q31 and 19q13.33 and two additional regions with max LOD that are approximately 2. The analyses were limited to variants residing within protein-coding genes, as defined by GENCODE v29 [34], of each linkage region in HyperGEN and GENOA TOPMed Freeze 8 WGS data.

Next, the variants were selected using a two-step approach. Step 1, let *LOD*_*j*_ represent the LOD score for the *j*^*th*^ family at the max LOD marker of a chromosomal region. We selected families with *LOD*_*j*_ > 0.1 after excluding parent-offspring pairs (e.g. family of two with mother-child or father-child), which are uninformative for linkage analysis. Prior simulations from our group have shown that the threshold of 0.1 for variant selection is optimal in association analysis [8]. We identified 35 families for 1q31, 18 families for 1q42, 20 families for 19q13.11 and 25 families for 19q13.33 with *LOD*_*j*_ > 0.1. SNPs or INDELs segregating at least twice in these families were selected. Step 2, let *MAC*_*ij*_ be the minor allele count for family *j* and variant *i* identified from step 1. For variant *i* in gene *x*, the correlation *r*_i_ between *MAC*_*ij*_ and *LOD*_*j*_ was calculated. When a portion of the variants in the linkage region contribute to linkage evidence, we expect that variants contributing to linkage evidence are more likely to have *r*_i_ to be positively correlated. For the variants in a gene *x*, their *r*_*i*_ were fitted a mixture of two Normal distributions using the mixtools R package. Then Fisher’s Discriminant Analysis was used to identify variants in which their correlation *r*_i_ is greater than the average of two component mean. Lastly, the union of variants selected by these two steps were included for association analysis. This process can be viewed as a weighting procedure of variants contributing to the observed linkage evidence.

The gene region is defined by Ensembl Variant Effect Predictor [35] as a part of the functional annotations curated by WGSA [36], which was provided by the TOPMed DCC. The variants selected for analysis were grouped into 2 sets using annotations: 1) functional coding variants that lead to an amino acid change and 2) remaining non-coding variants and synonymous variants located within the gene region and 10 kb upstream and downstream of each gene. Functional coding variants were limited to those with MAF < 5% and included splice region variant, start lost variant, stop lost/gained variant, missense variant, inframe deletions/insertions, exon loss variant (deletion of an exon), frameshift variant, initiator codon variant non-canonical start codon, and splice acceptor variant. The non-coding variants had a maximum MAF of 1% and were further examined for those with functional prediction scores [37, 38] (CADD-phred > 10, fathmmXF > 0.5). Within each coding and non-coding group, variants were aggregated by gene names. Variants located in multiple genes with overlapping positions were retained in each gene. We separately analyzed variants into two independent sets: set 1 includes coding variants with MAF < 5% and set 2 includes non-coding variants with MAF < 1%. We further combined set 1 and 2 variants (set 3) but required the set 2 variants with either CADD > 10 or fathmmXF > 0.5 [37, 38].

### Discovery association analyses with TOPMed WGS data

The focus of this study was performing gene-based association analyses in all four linkage regions for the three variant sets prioritized using linkage evidence with the GENESIS [24] R package. The majority of the analyses were completed on the High Performance Computing Cluster (HPCC) at Case Western Reserve University and parts of the trans-ancestry analysis were completed in Analysis Commons [39] on the cloud computing platform DNAnexus (https://www.dnanexus.com/) for computational efficiency. Discovery samples were stratified by ancestry (AA, EA, EAS, HA, Samoan) and both ancestry-specific and pooled trans-ancestry analyses were completed for SBP, DBP, and PP. A kinship matrix was constructed for each stratum and the trans-ancestry sample using the fourth-degree sparse kinship matrix provided by the TOPMed DCC. For each trait on each stratum, a null model was fitted using linear mixed model with the transformed phenotype residuals, covariates, and kinship matrix. Next, the three collapsed variant sets described previously were used to conduct gene-based association analysis using burden (Wald) test [40] and sequence kernel association test (SKAT) [41]. Variants were weighted using the default parameters dbeta [1, 25] to give more weight to the rarer variants. Bonferroni correction for the number of genes tested in each linkage region was used as a discovery significance threshold. After identifying top associated genes, we performed single SNP based association in order to identify individual variants contributing the gene-based association evidence. Single variant association analyses were completed using linear mixed model with GENESIS [24].

### Replication association analyses of unrelated samples in TOPMed-imputed UK Biobank

For the genes carried forward for replication analyses, we used the same gene collapsing groups to perform burden test and SKAT with TOPMed-imputed UK Biobank data in the GENESIS R package [24]. Single variant association analyses were only carried out for the top gene of interest, *RCN3*. Association analyses were performed without including a kinship matrix after removing individuals up to the 3rd degree of relatedness.

### Meta-analyses of TOPMed and UK Biobank

For the gene-based analyses, meta-analyses of European cohorts, African cohorts, and trans-ethnic cohorts from TOPMed and the UK Biobank were calculated using Fisher’s combined *p* value method. The trans-ethnic meta-analysis of TOPMed and UKB was also performed using Fisher’s method with 8 degrees of freedom to account for three UKB ancestry-specific analyses for individuals of European, African, and Asian ancestries. The exome-wide significance threshold (*p* < 2.5 × 10^− 6^) was used to determine genome-wide significance.

### Gene expression association analysis

Genotype-Tissue Expression (GTEx) expression quantitative trait loci (eQTL) gene expression matrices (GTEx V7 *cis*-eQTL) were downloaded from the GTEx Portal (https://www.gtexportal.org/home/datasets) and WGS data of 635 individuals were obtained from dbGaP phs000424.v7.p2. Tissue-specific gene expression association analyses were completed for genes of interest in 46 tissues and 2 cell lines. SKAT and burden test were completed in the software EPACTS [42] using both coding and non-coding variants in genes of interest identified from TOPMed (variant set 3). The residuals of the gene expression level were treated as the phenotype, after adjusting for sex, platform, PCs 1–3, and tissue-specific latent factors inferred by GTEx using the PEER method [43]. The analyzed variants were limited to variants replicated across studies, where we aggregated linkage-based selected functional coding variants and rare non-coding variants identified from HyperGEN and GENOA.

## Supplementary Information

**Additional file 1.** Supplemental Materials & Methods.

**Additional file 2: Table S1.** Characteristics of UK Biobank European samples.

**Additional file 3: Fig. S1.** TOPMed Freeze 8 phenotype distributions in African Americans.

**Additional file 4: Fig. S2.** TOPMed Freeze 8 phenotype distributions in European Americans.

**Additional file 5: Fig. S3.** TOPMed Freeze 8 phenotype distributions in East Asian/Asian Americans.

**Additional file 6: Fig. S4.** TOPMed Freeze 8 phenotype distributions in Hispanic Americans.

**Additional file 7: Fig. S5.** TOPMed Freeze 8 phenotype distributions in Samoans.

**Additional file 8.** Members of the Samoan Obesity, Lifestyle and Genetic Adaptations Study (OLaGA) Group.

**Additional file 9.** Members of the NHLBI Trans-Omics for Precision Medicine (TOPMed) Consortium.

## Data Availability

All the TOPMed datasets generated and/or analyzed during the current study are available in the dbGaP repository and instructions for data access can be found at https://www.nhlbiwgs.org/topmed-data-access-scientific-community. The current study includes datasets: phs000956, phs001211, phs001644, phs001624, phs001612, phs000954, phs001368, phs000951, phs001218, phs001345, phs000974, phs001217, phs001395, phs001293, phs000964, phs001416, phs001215, phs000972, phs001387, phs001237. The UK Biobank data is available in the UK Biobank repository: ukbiobank.ac.uk.

## Abbreviations

AA: African American
BP: Blood pressure
BMI: Body mass index
DBP: Diastolic blood pressure
DCC: Data coordinating center
EA: European American
EAS: East Asian
FBPP: Family Blood Pressure Program
GENESIS: GENetic EStimation and Inference in Structured samples
GENOA: Genetic Epidemiology Network of Arteriopathy
GTEx: Genotype-Tissue Expression
GWAS: Genome-wide association studies
HA: Hispanic American
HyperGEN: Hypertension Genetic Epidemiology Network
LD: Linkage disequilibrium
LOD: Logarithm of odds
MAC: Minor allele count
MAF: Minor allele frequency
MLOD: MLOD
NHLBI: National Heart, Lung, and Blood Institute
PC: Principal component
PP: Pulse pressure
QC: Quality control
R-INT: Rescaled inverse normal transformation
SBP: Systolic blood pressure
SKAT: Sequence kernel association test
SNP: Single nucleotide polymorphism
TOPMed: Trans-Omics for Precision Medicine
UKB: UK Biobank
WGS: Whole genome sequencing
