# Supplementary material for: Rare coding variants in RCN3 are associated with blood pressure
Source: BMC Genomics. 2022 Feb 19;23:148. doi: 10.1186/s12864-022-08356-4 (PMC8858539; doi:10.1186/s12864-022-08356-4)
Supplement: Supplementary file 1 — Additional file 1. Supplemental Materials & Methods. [file 12864_2022_8356_MOESM1_ESM.docx]

**Supplemental Materials & Methods**

**Descriptions of studies included**

**Amish:** The Genetics of Cardiometabolic Health in the Amish is a family-based study of relatively healthy Old Order Amish individuals who were recruited into various community-based studies largely focused on cardiometabolic health. Blood pressure was measured by standard sphygmomanometer. The field center variable is based on different studies that the participants were enrolled in, not different data collection centers. Study 1 was a short-term intervention that recruited relatively healthy participants. Study 2 was a longevity study that recruited probands 90 years or older and their family members and family members’ spouses. Study 3 was a community-based “wellness” study that recruits all Old Order Amish.

**ARIC:** The Atherosclerosis Risk in Communities (ARIC) study is a prospective population-based cohort study designed to investigate the etiology and predictors of cardiovascular disease, and has been described in detail previously (PMID: 2646917). Briefly, participants aged 45 to 64 years at baseline were recruited from four communities: Forsyth County, North Carolina; Jackson, Mississippi; Minneapolis, Minnesota; and Washington County, Maryland. A total of 15,792 individuals, predominantly of European and African ancestry, participated in the baseline examination in 1987-1989, with three additional triennial follow-up examinations and a fifth exam in 2011-2013. Blood pressure was measured by random zero sphygmomanometer.

**BioMe:** The Institute for Personalized Medicine BioMe Biobank, founded in September 2007, is an ongoing, broadly-consented electronic health record-linked clinical care biobank that enrolls participants non-selectively from the Mount Sinai Medical Center patient population. For the TOPMed Program, adult (>18 years of age) coronary artery disease (CAD), atrial fibrillation, and chronic obstructive pulmonary disease (COPD) cases and controls were included. A Case-Definition-Algorithm (CDA), incorporating International Classification of Diseases (ICD) codes and Current Procedural Terminology (CPT) codes, was used to identify individuals with CAD along with suitable controls. A previously validated COPD CDA (PMID: 28507288), incorporating ICD codes and medication history, was used to identify individuals with COPD along with suitable controls. Finally, a sample was selected from BioMe participants with atrial fibrillation, all of African ancestry, identified using ICD or CPT codes. Blood pressure was measured by mixed sphygmomanometer types used in medical care.

**CARDIA:** The Coronary Artery Risk Development in Young Adults (CARDIA) study is a multi-center prospective study. There were 5,115 participants aged 18 to 30 at baseline. The cohort was equally distributed by age group (18-24; 25-30), sex, race (black and white), and education (less than or equivalent to high school, more than high school). Four field centers across the US were included: Birmingham, AL; Chicago, IL; Minneapolis, MN; Oakland, CA. Participants from the Birmingham, Chicago, and Minneapolis centers were recruited from the total community or from selected census tracts. Participants from the Oakland center were randomly recruited from the Kaiser-Permanente health plan membership. Blood pressure data used in this study were harmonized by the TOPMed DCC, for which the strategy is described on https://www.nhlbiwgs.org/dcc-harmonized-phenotypes.

**CFS:** The Cleveland Family Study (CFS) is a family-based cohort. Index probands (n=275) were recruited from 3 area hospital sleep labs if they had a confirmed diagnosis of sleep apnea and at least 2 first-degree relatives available to be studied. In the first 5 years of the study, neighborhood control probands (n=87) with at least 2 living relatives available for study were selected at random from a list provided by the index family and also studied. All available first-degree relatives and spouses of the case and control probands also were recruited. Second-degree relatives, including half-sibs, aunts, uncles and grandparents, were also included if they lived near the first-degree relatives (cases or controls), or if the family had been found to have two or more relatives with sleep apnea. Blood was sampled and DNA isolated for participants seen in the last two exam cycles (n=1447). The sample, which is enriched with individuals with sleep apnea, also contains a high prevalence of individuals with sleep apnea-related traits, including: obesity, impaired glucose tolerance, and hypertension. Blood pressure was measured by calibrated sphygmomanometer. Unlike other studies, the harmonized BP data reported last visit values (per usual CFS procedures) due to improved phenotyping at the general clinical research center visit.

**CHS:** The Cardiovascular Health Study (CHS) is a longitudinal cohort study of cardiovascular disease (CVD) and cardiovascular risk factors in adults. The data requested for this phenotype file are from the baseline visit: 1989-1990 for the predominately Caucasian original cohort (cohort 1), and 1992-1993 for the African-American cohort (cohort 2).

CHS is comprised of four Field Centers, or clinics, numbered 3-6 in the CHS data:

3) Wake Forest University School of Medicine, Forsyth County, Winston-Salem, NC

4) University of California, Davis, Sacramento County, Sacramento, CA

5) Johns Hopkins University, Washington County, Hagerstown, MD

6) University of Pittsburgh, Pittsburgh, PA

Because there is potential for data to vary by Field Center, Field Center (FC) is included as a covariate in our analyses.

Sitting blood pressure was measured in the right arm of seated participants after a five-minute rest using an appropriately-sized cuff and a Hawksley random zero sphygmomanometer, model 7076 (Hawksley and Sons Limited, Sussex, England). The average of two measurements of the first (systolic) and fifth (diastolic) Korotkoff sounds was used for analysis (PMID: 1669507). Blood pressure data used in this study were harmonized by the TOPMed DCC, for which the strategy is described on https://www.nhlbiwgs.org/dcc-harmonized-phenotypes.

**FHS:** The Framingham Heart Study (FHS) is a prospective family-based study. Participants were selected through population of three generations of the Framingham Heart Study. The oldest (original) cohort were selected through city-wide invitation in 1948 for middle-aged adults those provided complete information on health-related information and attended on-site clinical measurements. Children and their spouse of the original cohort were invited to the study started in 1971 that composed the OFFspring cohort. Children of the offspring cohort began the third-generation cohort study in 2002. Blood pressure was measured by desktop baumanometer (E98169).

**GeneSTAR:** The Genetic Study of Atherosclerosis Risk (GeneSTAR) is a family-based study. GeneSTAR began in 1982 as the Johns Hopkins Sibling and Family Heart Study, a prospective longitudinal family-based study conducted originally in healthy adult siblings of people with documented early onset coronary disease under 60 years of age. Commencing in 2003, the siblings, their offspring, and the coparent of the offspring who were free of cardiovascular disease participated in a 2-week trial of aspirin 81 mg/day with pre and post ex vivo platelet function assessed using multiple agonists. Of the 2142 participants with complete measures of platelet function, 1786 were selected for TOPMed based on (1) participation in later GeneSTAR studies and (2) largest family size. Blood pressure was measured by mercury or aneroid sphygmomanometer.

**GENOA:** The Genetic Epidemiology Network of Arteriopathy (GENOA) study consists of hypertensive sibships that were recruited for linkage and association studies in order to identify genes that influence blood pressure and its target organ damage (PMID: 15121494). In the initial phase of the GENOA study (Phase I: 1996-2001), all members of sibships containing ≥ 2 individuals with essential hypertension clinically diagnosed before age 60 were invited to participate, including both hypertensive and normotensive siblings. A total of 1,583 non-Hispanic whites from Rochester, MN, and 1,854 African Americans from Jackson, MS, were examined. In the second phase of the GENOA study (Phase II: 2000-2004), 1,239 non-Hispanic white and 1,482 African American participants were successfully re-recruited to measure potential target organ damage due to hypertension. Following Phase II, several GENOA ancillary studies were conducted, including the Coronary Artery Calcification (CAC) Study (2009-2011).

Sitting systolic blood pressure (SBP) (mmHg) and diastolic blood pressure (DBP) (mmHg) were measured three times with a random zero sphygmomanometer. The average of the last two measurements at Phase I was used in this study. For subjects taking any anti-hypertensive (BP lowering) medications, 15 mmHg was added to SBP and 10 mmHg was added to DBP. A physician specializing in hypertension reviewed all medications and made the final determination of whether a medication was considered an anti-hypertensive.

**GenSalt:** The GenSalt study has a family feeding-study design. A community-based BP screening was conducted among persons aged 18–60 years to identify potential probands and their families. Probands with untreated prehypertension or stage-1 hypertension and their spouses, siblings, and offspring were recruited as volunteers for the study. Those who had stage-2 hypertension, secondary hypertension, a history of clinical cardiovascular disease or diabetes, used antihypertensive medications, or were pregnant, heavy alcohol drinkers, or currently on a low-sodium diet were excluded from the study. Among the 1,906 eligible participants from 633 families, 1,872 (98.2%) agreed to participate and were included in the TOPMed WGS study. Blood pressure was measured by standard mercury sphygmomanometer.

**HCHS_SOL:** The Hispanic Community Health Study – Study of Latinos (HCHS_SOL) is a multicenter prospective cohort recruited in 2008-2011. Participants between 18 to 74 years old (N=16,415) were recruited from four US cities. One-third of participants were 18-45 years at baseline (undersampled) and two-thirds of participants were over 45 years at baseline (oversampled). Genetic ancestries of participants include Puerto Rican (15.8%), Dominican (8.5%), Cuban (13.4%), Mexican (38%), Central American (10%), South American (6.2%), and others (8.7%). Blood pressure data used in this study were harmonized by the TOPMed DCC, for which the strategy is described on https://www.nhlbiwgs.org/dcc-harmonized-phenotypes.

**HyperGen:** The Hypertension Genetic Epidemiology Network (HyperGEN) study is one of the four networks in the Family Blood Pressure Program (FBPP) supported by the National Heart, Lung, and Blood Institute to identify genetic contributors to hypertension. HyperGEN is a family-based study with a sib-pair design. Hypertensive African American sibships were recruited from population-based cohorts in Forsyth County, NC, and from the community-at-large in Birmingham, AL, from 1995 to 2000. Sibling pairs with onset of hypertension before age 60 were recruited in the first phase. The study was later extended to other siblings and the offspring of the hypertensive probands who were unmedicated adults. Blood pressure was measured by automated Dinamap devices (model 1846 SX/P, Critikon, Tampa, FL).

**JHS:** The Jackson Heart Study (JHS) is a large, community-based, observational study whose participants were recruited from urban and rural areas of the three counties (Hinds, Madison and Rankin) that make up the Jackson, Mississippi metropolitan statistical area (MSA). Participants were enrolled from each of 4 recruitment pools: random, 17%; volunteer, 22%; currently enrolled in the Atherosclerosis Risk in Communities (ARIC) Study, 30% and secondary family members, 31%. Recruitment was limited to non-institutionalized adult African Americans 35-84 years old, except in the family cohort where those 21 to 34 years of age were eligible. The final cohort of 5,306 participants includes 6.59% of all African American Jackson MSA residents aged 35-84 (N-76,426, US Census 2000). Out of the 5,306 eligible participants, 3,406 (64.2%) agreed to participate in genetic analyses, passed quality control, and were included in the TOPMed WGS study. Blood pressure was measured by random zero sphygmomanometer.

**MESA:** The Multi-Ethnic Study of Atherosclerosis (MESA) is a study of characteristics of subclinical cardiovascular disease, the risk factors that predict progression to clinically overt cardiovascular disease, and the risk factors that predict progression of subclinical disease. MESA includes a diverse, population-based sample of 6,814 asymptomatic men and women aged 45-84 from six field centers across the US. Ancestral composition consists of 38% white, 28% African-American, 22% Hispanic, and 12% Asian (predominantly of Chinese descent). The MESA design and recruitment methods are described in detail elsewhere (PMID: 12397006). Resting seated BP was measured three times in the right arm using a Dinamap model Pro 100 automated sphygmomanometer (Critikon, Tampa, FL) and the average of the last two measurements was used in the analysis (PMID: 17070420).

MESA has several ancillary studies, including the MESA Family cohort, which is a part of the African American Coronary Artery Calcification project in TOPMed.

**Samoan:** The Samoan Adiposity Study is a population-based cohort. Over 3,400 individuals ages 25-65 years were recruited in 2010 from 33 villages from all census regions of the nation, which is experiencing economic development and the nutrition transition. Eligibility was based on self-report of having four Samoan grandparents, not being pregnant, and not having severe physical impairment which would prohibit collection of anthropometric, biomarker and questionnaire measures, nor cognitive impairment which would not allow informed consent about the genetic purposes of the study. Blood pressure was measured by Omron digital blood pressure monitor. Phenotype data used in this study were harmonized by the TOPMed DCC, for which the strategy is described on https://www.nhlbiwgs.org/dcc-harmonized-phenotypes.

**SAFS:** The San Antonio Family Heart Study, part of the San Antonio Family Studies (SAFS), is a complex family-based mixed longitudinal study designed to identify low frequency or rare variants influencing susceptibility to cardiovascular disease. It includes whole genome sequence information from 2,590 individuals in large Mexican American pedigrees from San Antonio, TX. The systolic (first phase) and diastolic (fifth phase) blood pressures were measured to the nearest even digit using a random-zero sphygmomanometer (Hawksley-Gelman) on the right arm of the seated participant. Three readings were recorded for each individual, and the subject's blood pressure was defined as the average of the second and third readings (PMID: 8901667).

**THRV:** The THRV-TOPMed study consists of three participating cohorts: The SAPPHIRe Family Cohort, TSGH (Tri-Service General Hospital, a hospital-based cohort), and TCVGH (Taichung Veterans General Hospital, another hospital-based cohort), all based in Taiwan. For each cohort, the study data was collected in a variety of ways, including questionnaires, electronic medical records, and physical examination (through detailed protocols). Blood pressure was collected using Dinamap monitor model 1846 SX.

For SAPPHIRe, eligible families were sent detailed questionnaires beforehand on medical history, family history, age, sex, physical activity and drinking habits. Subjects were requested to fast for 12 hours overnight, and to attend the clinical study site between 8-9am. Upon arrival, blood pressure was measured according to approved core protocol. As long as the subject continued to meet eligibility criteria, fasting blood was drawn immediately. After this, selected physical measures were also conducted including body weight, height and waist circumference. The pre-sent questionnaire was collected and incomplete questions resolved at the time of the clinic visit by the SAPPHIRe staff. All subjects underwent Oral Glucose Tolerance test (OGTT), and some were evaluated for insulin resistance.

General blood chemistries were performed by central laboratory of SAPPHIRe, Taiwan, which was situated at National Taiwan University at the time. Certain items such as insulin and glucose were assayed by selected centralized Family Blood Pressure laboratories in the United states, specifically by General Clinical Research Center Laboratory at Stanford University, Medical Center.

The TSGH and TCVGH hospital-based cohorts were recruited following comparable inclusion/exclusion criteria. TCVGH utilized extensive electronic medical records (EMR) system, thus all data were extracted from the EMR of the subjects recruited either as inpatients, or from outpatient clinics. Although TSGH also has EMR system, it recruited their subjects as traditional study cohorts do, using questionnaires for information regarding medical history, family history, age, sex, physical activity and drinking habits. Blood was drawn in their metabolic research ward, and various chemistries were assessed by their research laboratories.

**WHI:** The Women’s Health Initiative (WHI) is a population-based cohort study in postmenopausal women in which case-control samples were selected for TOPMed. Sample include 5929 cases and 5171 controls. The cases had Ischemic or Hemorrhagic strokes or VTE. Controls were dbGaP eligible, with DNA already extracted and needed to have at least 5 biomarkers (LDL, HDL, Triglycerides, Glucose, Insulin, CRP and/or Creatinine). Controls were matched based on age at baseline and HRT participation. We looked at the 3 outcomes by race and whether DNA was available or needed to be extracted. Subjects eligible for controls were dbGaP eligible, no history of stroke or VTE and needed to have DNA available. The controls were selected to match the number of cases when they were stratified by age and HRT participation. Controls were selected within cells of age strata and race to match cases. Preference was given to samples with the greatest number of biomarkers; most cells had enough subjects with all 7 of the biomarkers (LDL, HDL, INSU, GLUC, CREA, CRP and TRI). If there were not enough with 7 biomarkers, we looked at cells with fewer biomarkers until enough subjects were selected. Since there is a relationship between the selection criteria and blood pressure, it was recommended to analyze VTE cases, stroke cases, and controls separately. We added a case-control variable in the model when calculating the residuals. Blood pressure was measured by conventional mercury sphygmomanometer.
