# Supplementary material for: Rare coding variants in RCN3 are associated with blood pressure
Source: BMC Genomics. 2022 Feb 19;23:148. doi: 10.1186/s12864-022-08356-4 (PMC8858539; doi:10.1186/s12864-022-08356-4)
Supplement: Supplementary file 2 — Additional file 2: Table S1. Characteristics of UK Biobank European samples. [file 12864_2022_8356_MOESM2_ESM.docx]

**Table S1.** Characteristics of UK Biobank European samples

|  | **European ancestry** | **African ancestry** | **Asian ancestry** |
| --- | --- | --- | --- |
| **Females** | 208,114 (53.8%) | 3,913 (56.4%) | 4,767 (48.8%) |
| **Age** | 38-73 (median: 58) | 39-70 (median: 51) | 40-72 (median: 53) |
| **BMI** | 12.12-74.68 (median: 26.70) | 16.15-68.13 (median: 28.65) | 14.87-60 (median: 26.14) |
| **Take anti-hypertensive medication** | 38,363 | 1,326 | 1,006 |
| **Ethnic subgroup** | 439 White  361,509 British  10,577 Irish  14,288 any other white background | 24 Black  3,841 Caribbean  2,968 African  104 any other black background | 39 Asian  5,057 Indian  1,517 Pakistani  205 Bangladeshi  1,600 any other Asian background  1,354 Chinese |
