# Supplementary figures and images for: Rare coding variants in RCN3 are associated with blood pressure
Source: BMC Genomics. 2022 Feb 19;23:148. doi: 10.1186/s12864-022-08356-4 (PMC8858539; doi:10.1186/s12864-022-08356-4)

**Figure S1.** TOPMed Freeze 8 phenotype distributions in African Americans


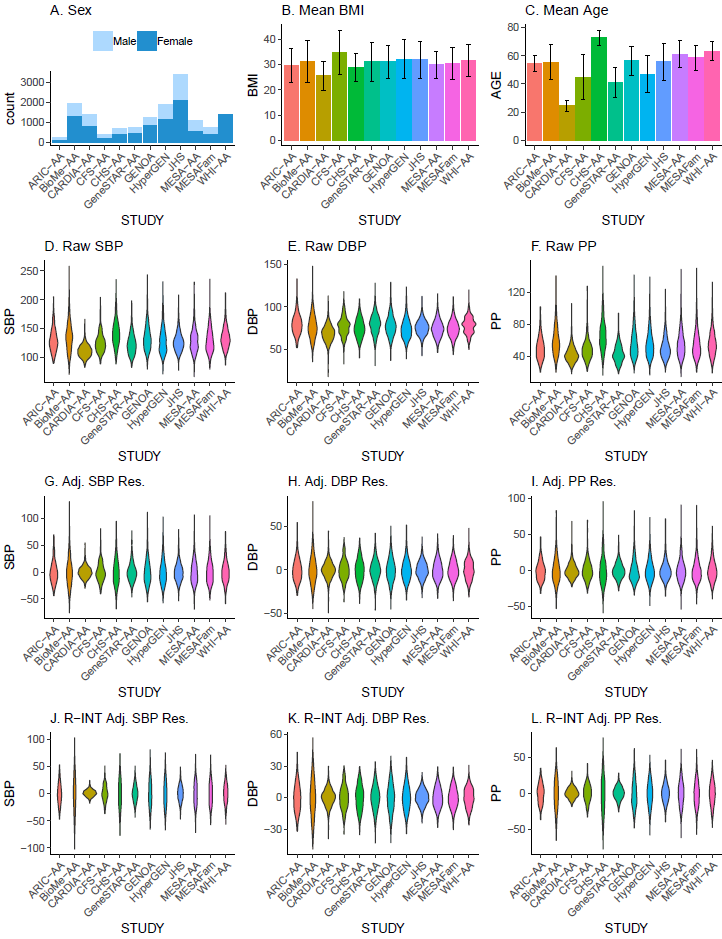

Supplement: Supplementary file 3 — Additional file 3: Fig. S1. TOPMed Freeze 8 phenotype distributions in African Americans. [file 12864_2022_8356_MOESM3_ESM.docx]

**Figure S2.** TOPMed Freeze 8 phenotype distributions in European Americans


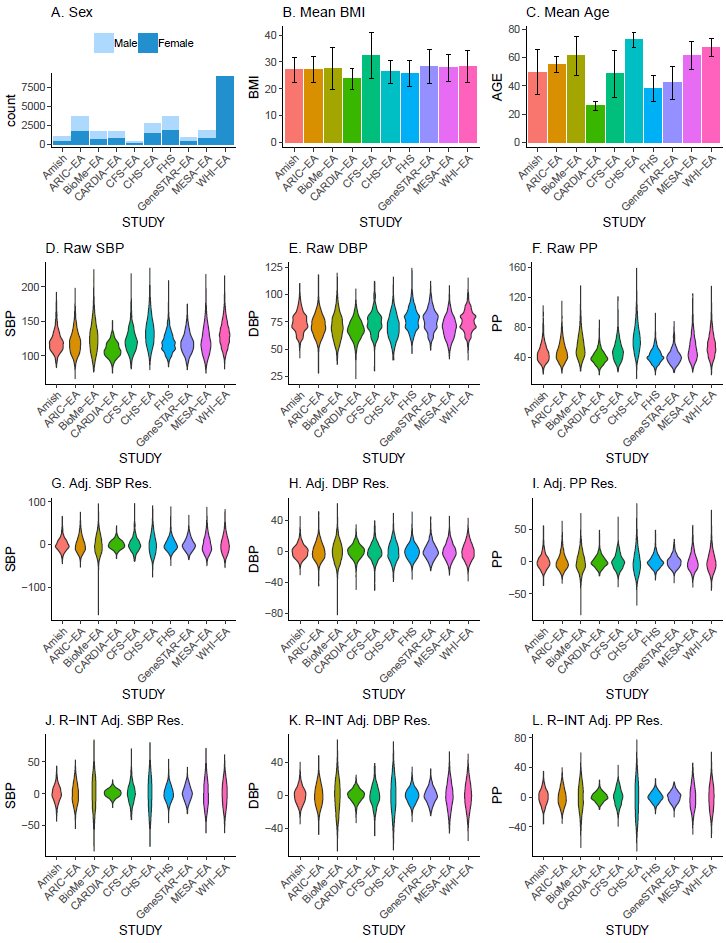

Supplement: Supplementary file 4 — Additional file 4: Fig. S2. TOPMed Freeze 8 phenotype distributions in European Americans. [file 12864_2022_8356_MOESM4_ESM.docx]

**Figure S3.** TOPMed Freeze 8 phenotype distributions in East Asian/Asian Americans


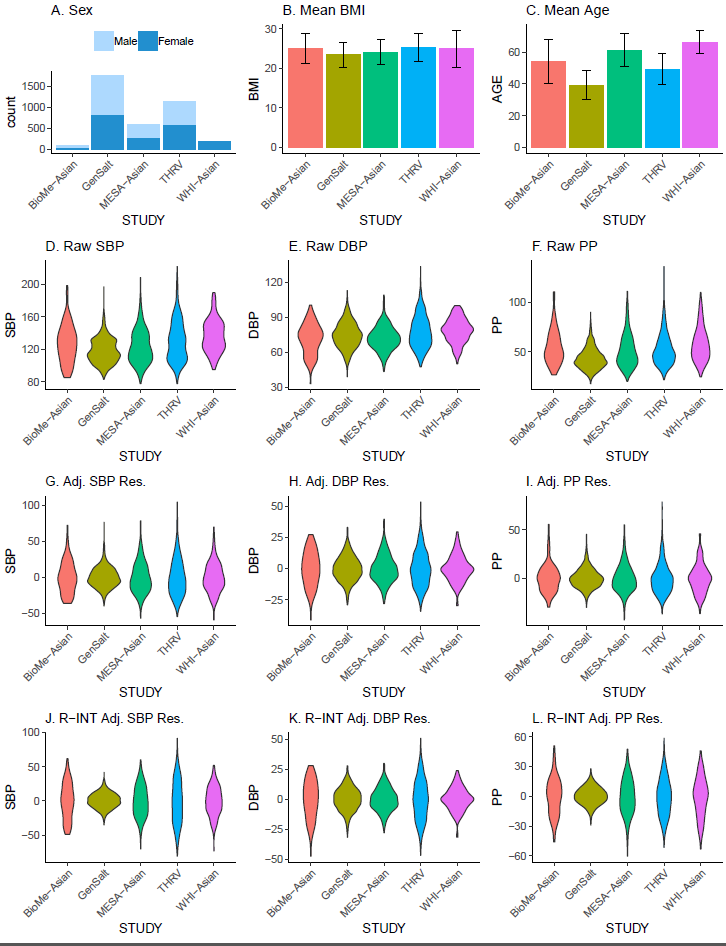

Supplement: Supplementary file 5 — Additional file 5: Fig. S3. TOPMed Freeze 8 phenotype distributions in East Asian/Asian Americans. [file 12864_2022_8356_MOESM5_ESM.docx]

**Figure S5.** TOPMed Freeze 8 phenotype distributions in Samoans


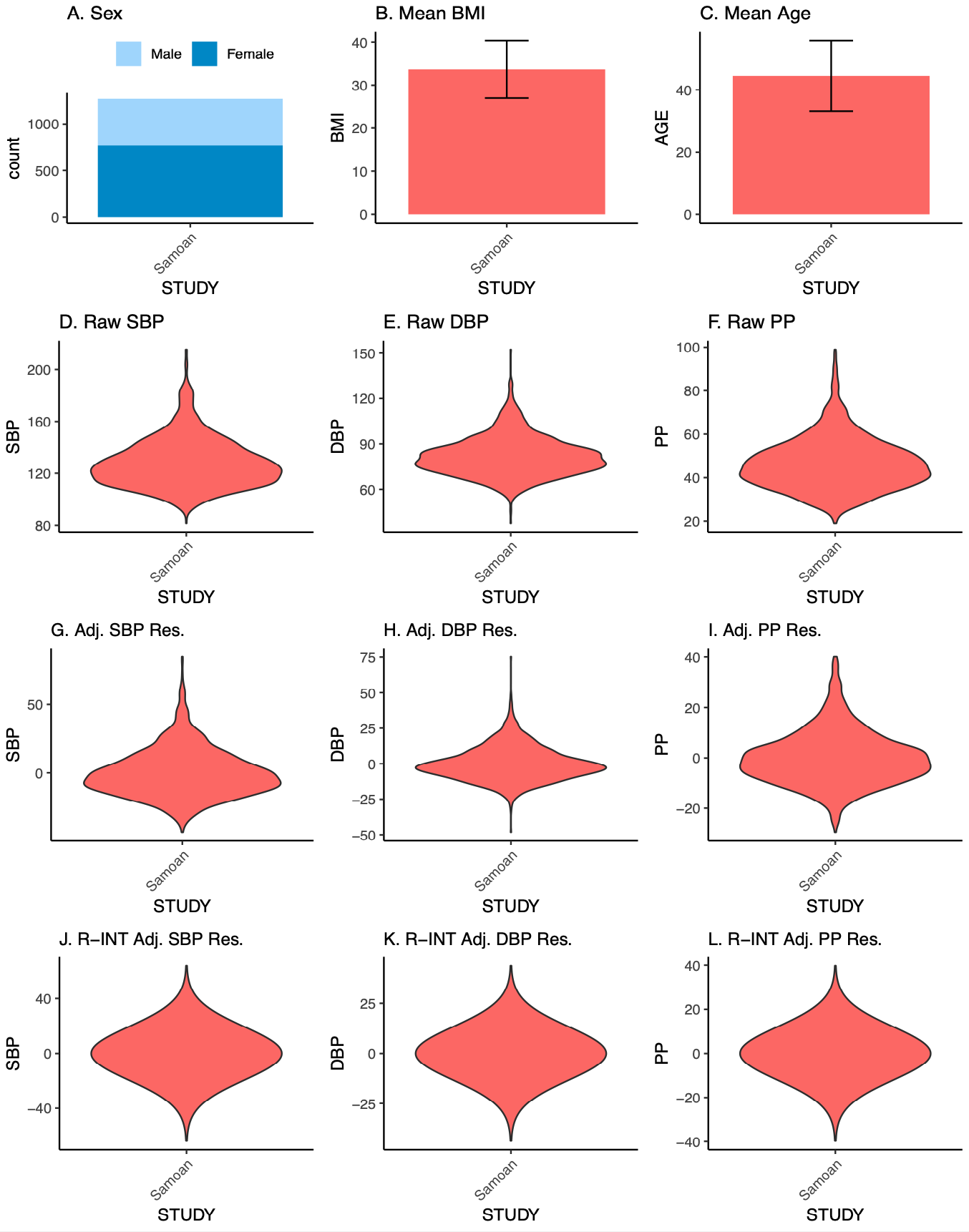

Supplement: Supplementary file 7 — Additional file 7: Fig. S5. TOPMed Freeze 8 phenotype distributions in Samoans. [file 12864_2022_8356_MOESM7_ESM.docx]
