# Supplementary material for: Rare coding variants in RCN3 are associated with blood pressure
Source: BMC Genomics. 2022 Feb 19;23:148. doi: 10.1186/s12864-022-08356-4 (PMC8858539; doi:10.1186/s12864-022-08356-4)
Supplement: Supplementary file 6 — Additional file 6: Fig. S4. TOPMed Freeze 8 phenotype distributions in Hispanic Americans. [file 12864_2022_8356_MOESM6_ESM.docx]

**Figure S4.** TOPMed Freeze 8 phenotype distributions in Hispanic Americans
