# Supplementary material for: Rare coding variants in RCN3 are associated with blood pressure
Source: BMC Genomics. 2022 Feb 19;23:148. doi: 10.1186/s12864-022-08356-4 (PMC8858539; doi:10.1186/s12864-022-08356-4)
Supplement: Supplementary file 8 — Additional file 8. Members of the Samoan Obesity, Lifestyle and Genetic Adaptations Study (OLaGA) Group. [file 12864_2022_8356_MOESM8_ESM.docx]

**Members of the Samoan Obesity, Lifestyle and Genetic Adaptations Study (OLaGA) Group**

Ranjan Deka, Dept. of Environmental Health, University of Cincinnati, Cincinnati, Ohio, USA

Nicola L. Hawley, Dept. of Chronic Disease Epidemiology, Yale University, New Haven, Connecticut, USA

Stephen T McGarvey, Dept. of Epidemiology and International Health Institute, and Dept. of Anthropology, Brown University, Providence, Rhode Island, USA

Ryan L Minster, Dept. of Human Genetics, University of Pittsburgh, Pittsburg, Pennsylvania, USA

Take Naseri, Ministry of Health, Government of Samoa

Muagututi‘a Sefuiva Reupena, Lutia I Puava Ae Mapu I Fagalele

Daniel E. Weeks, Depts. of Human Genetics and Biostatistics, University of Pittsburgh, Pittsburg, Pennsylvania, USA
