# Supplementary material for: Rare coding variants in RCN3 are associated with blood pressure
Source: BMC Genomics. 2022 Feb 19;23:148. doi: 10.1186/s12864-022-08356-4 (PMC8858539; doi:10.1186/s12864-022-08356-4)
Supplement: Supplementary file 9 — Additional file 9. Members of the NHLBI Trans-Omics for Precision Medicine (TOPMed) Consortium. [file 12864_2022_8356_MOESM9_ESM.docx]

**Members of the NHLBI Trans-Omics for Precision Medicine (TOPMed) Consortium**

| **Name** | **Institution(s)** | **Primary Department** | **Institution City** | **Institution State** | **Zip Code** | **Country** |
| --- | --- | --- | --- | --- | --- | --- |
| Abe, Namiko | New York Genome Center |  | New York | New York | 10013 | US |
| Abecasis, Gonçalo | University of Michigan |  | Ann Arbor | Michigan | 48109 | US |
| Aguet, Francois | Broad Institute |  | Cambridge | Massachusetts | 02142 | US |
| Albert, Christine | Cedars Sinai |  | Boston | Massachusetts | 02114 | US |
| Almasy, Laura | Children's Hospital of Philadelphia, University of Pennsylvania |  | Philadelphia | Pennsylvania | 19104 | US |
| Alonso, Alvaro | Emory University |  | Atlanta | Georgia | 30322 | US |
| Ament, Seth | University of Maryland |  | Baltimore | Maryland | 21201 | US |
| Anderson, Peter | University of Washington |  | Seattle | Washington | 98195 | US |
| Anugu, Pramod | University of Mississippi |  | Jackson | Mississippi | 38677 | US |
| Applebaum-Bowden, Deborah | National Institutes of Health |  | Bethesda | Maryland | 20892 | US |
| Ardlie, Kristin | Broad Institute |  | Cambridge | Massachusetts | 02142 | US |
| Arking, Dan | Johns Hopkins University |  | Baltimore | Maryland | 21218 | US |
| Arnett, Donna K | University of Kentucky |  | Lexington | Kentucky | 40506 | US |
| Ashley-Koch, Allison | Duke University |  | Durham | North Carolina | 27708 | US |
| Aslibekyan, Stella | University of Alabama |  | Birmingham | Alabama | 35487 | US |
| Assimes, Tim | Stanford University |  | Stanford | California | 94305 | US |
| Auer, Paul | University of Wisconsin Milwaukee |  | Milwaukee | Wisconsin | 53211 | US |
| Avramopoulos, Dimitrios | Johns Hopkins University |  | Baltimore | Maryland | 21218 | US |
| Ayas, Najib | Providence Health Care | Medicine | Vancouver |  |  | CA |
| Barnard, John | Cleveland Clinic |  | Cleveland | Ohio | 44195 | US |
| Barnes, Kathleen | University of Colorado Anschutz Medical Campus |  | Aurora | Colorado | 80045 | US |
| Barr, R. Graham | Columbia University |  | New York | New York | 10027 | US |
| Barron-Casella, Emily | Johns Hopkins University |  | Baltimore | Maryland | 21218 | US |
| Barwick, Lucas | The Emmes Corporation | LTRC | Rockville | Maryland | 20850 | US |
| Beaty, Terri | Johns Hopkins University |  | Baltimore | Maryland | 21218 | US |
| Beck, Gerald | Cleveland Clinic | Quantitative Health Sciences | Cleveland | Ohio | 44195 | US |
| Becker, Diane | Johns Hopkins University | Medicine | Baltimore | Maryland | 21218 | US |
| Becker, Lewis | Johns Hopkins University |  | Baltimore | Maryland | 21218 | US |
| Beer, Rebecca | National Heart, Lung, and Blood Institute, National Institutes of Health |  | Bethesda | Maryland | 20892 | US |
| Beitelshees, Amber | University of Maryland |  | Baltimore | Maryland | 21201 | US |
| Benjamin, Emelia | Boston University, Massachusetts General Hospital | Boston University School of Medicine | Boston | Massachusetts | 02114 | US |
| Benos, Takis | University of Pittsburgh |  | Pittsburgh | Pennsylvania | 15260 | US |
| Bezerra, Marcos | Fundação de Hematologia e Hemoterapia de Pernambuco - Hemope |  | Recife |  | 52011-000 | BR |
| Bielak, Larry | University of Michigan |  | Ann Arbor | Michigan | 48109 | US |
| Bis, Joshua | University of Washington | Cardiovascular Health Research Unit, Department of Medicine | Seattle | Washington | 98195 | US |
| Blackwell, Thomas | University of Michigan |  | Ann Arbor | Michigan | 48109 | US |
| Blangero, John | University of Texas Rio Grande Valley School of Medicine | Human Genetics | Brownsville | Texas | 78520 | US |
| Boerwinkle, Eric | University of Texas Health at Houston |  | Houston | Texas | 77225 | US |
| Bowden, Donald W. | Wake Forest Baptist Health | Department of Biochemistry | Winston-Salem | North Carolina | 27157 | US |
| Bowler, Russell | National Jewish Health | National Jewish Health | Denver | Colorado | 80206 | US |
| Brody, Jennifer | University of Washington |  | Seattle | Washington | 98195 | US |
| Broeckel, Ulrich | Medical College of Wisconsin |  | Milwaukee | Wisconsin | 53226 | US |
| Broome, Jai | University of Washington |  | Seattle | Washington | 98195 | US |
| Brown, Deborah | University of Texas Health at Houston | Pediatrics | Houston | Texas | 77030 | US |
| Bunting, Karen | New York Genome Center |  | New York | New York | 10013 | US |
| Burchard, Esteban | University of California, San Francisco |  | San Francisco | California | 94143 | US |
| Bustamante, Carlos | Stanford University | Biomedical Data Science | Stanford | California | 94305 | US |
| Buth, Erin | University of Washington | Biostatistics | Seattle | Washington | 98195 | US |
| Cade, Brian | Brigham & Women's Hospital | Brigham and Women's Hospital | Boston | Massachusetts | 02115 | US |
| Cardwell, Jonathan | University of Colorado at Denver |  | Denver | Colorado | 80204 | US |
| Carey, Vincent | Brigham & Women's Hospital |  | Boston | Massachusetts | 02115 | US |
| Carrier, Julie | University of Montreal |  |  |  |  | US |
| Carty, Cara | Washington State University |  | Pullman | Washington | 99164 | US |
| Casaburi, Richard | University of California, Los Angeles |  | Los Angeles | California | 90095 | US |
| Casas Romero, Juan P | Brigham & Women's Hospital |  |  |  |  | US |
| Casella, James | Johns Hopkins University |  | Baltimore | Maryland | 21218 | US |
| Castaldi, Peter | Brigham & Women's Hospital | Medicine | Boston | Massachusetts | 02115 | US |
| Chaffin, Mark | Broad Institute |  | Cambridge | Massachusetts | 02142 | US |
| Chang, Christy | University of Maryland |  | Baltimore | Maryland | 21201 | US |
| Chang, Yi-Cheng | National Taiwan University |  | Taipei |  | 10617 | TW |
| Chasman, Daniel | Brigham & Women's Hospital | Division of Preventive Medicine | Boston | Massachusetts | 02215 | US |
| Chavan, Sameer | University of Colorado at Denver |  | Denver | Colorado | 80204 | US |
| Chen, Bo-Juen | New York Genome Center |  | New York | New York | 10013 | US |
| Chen, Wei-Min | University of Virginia |  | Charlottesville | Virginia | 22903 | US |
| Chen, Yii-Der Ida | Lundquist Institute |  | Torrance | California | 90502 | US |
| Cho, Michael | Brigham & Women's Hospital |  | Boston | Massachusetts | 02115 | US |
| Choi, Seung Hoan | Broad Institute |  | Cambridge | Massachusetts | 02142 | US |
| Chuang, Lee-Ming | National Taiwan University | National Taiwan University Hospital | Taipei |  | 10617 | TW |
| Chung, Mina | Cleveland Clinic | Cleveland Clinic | Cleveland | Ohio | 44195 | US |
| Chung, Ren-Hua | National Health Research Institute Taiwan |  | Miaoli County |  | 350 | TW |
| Clish, Clary | Broad Institute | Metabolomics Platform | Cambridge | Massachusetts | 02142 | US |
| Comhair, Suzy | Cleveland Clinic | Immunity and Immunology | Cleveland | Ohio | 44195 | US |
| Conomos, Matthew | University of Washington | Biostatistics | Seattle | Washington | 98195 | US |
| Cornell, Elaine | University of Vermont |  | Burlington | Vermont | 05405 | US |
| Correa, Adolfo | University of Mississippi | Medicine | Jackson | Mississippi | 38677 | US |
| Crandall, Carolyn | University of California, Los Angeles |  | Los Angeles | California | 90095 | US |
| Crapo, James | National Jewish Health |  | Denver | Colorado | 80206 | US |
| Cupples, L. Adrienne | Boston University | Biostatistics | Boston | Massachusetts | 02115 | US |
| Curran, Joanne | University of Texas Rio Grande Valley School of Medicine |  | Brownsville | Texas | 78520 | US |
| Curtis, Jeffrey | University of Michigan |  | Ann Arbor | Michigan | 48109 | US |
| Custer, Brian | Vitalant Research Institute |  | San Francisco | California | 94118 | US |
| Damcott, Coleen | University of Maryland |  | Baltimore | Maryland | 21201 | US |
| Darbar, Dawood | University of Illinois at Chicago |  | Chicago | Illinois | 60607 | US |
| Das, Sayantan | University of Michigan |  | Ann Arbor | Michigan | 48109 | US |
| David, Sean | University of Chicago |  | Chicago | Illinois | 60637 | US |
| Davis, Colleen | University of Washington |  | Seattle | Washington | 98195 | US |
| Daya, Michelle | University of Colorado at Denver |  | Denver | Colorado | 80204 | US |
| de Andrade, Mariza | Mayo Clinic | Health Sciences Research | Rochester | Minnesota | 55905 | US |
| de las Fuentes, Lisa | Washington University in St Louis | Department of Medicine, Cardiovascular Division | St. Louis | Missouri | 63110 | US |
| DeBaun, Michael | Vanderbilt University |  | Nashville | Tennessee | 37235 | US |
| Deka, Ranjan | University of Cincinnati |  | Cincinnati | Ohio | 45220 | US |
| DeMeo, Dawn | Brigham & Women's Hospital |  | Boston | Massachusetts | 02115 | US |
| Devine, Scott | University of Maryland |  | Baltimore | Maryland | 21201 | US |
| Duan, Qing | University of North Carolina |  | Chapel Hill | North Carolina | 27599 | US |
| Duggirala, Ravi | University of Texas Rio Grande Valley School of Medicine |  | Edinburg | Texas | 78539 | US |
| Durda, Jon Peter | University of Vermont |  | Burlington | Vermont | 05405 | US |
| Dutcher, Susan | Washington University in St Louis |  | St Louis | Missouri | 63130 | US |
| Eaton, Charles | Brown University |  | Providence | Rhode Island | 02912 | US |
| Ekunwe, Lynette | University of Mississippi |  | Jackson | Mississippi | 38677 | US |
| El Boueiz, Adel | Harvard University | Channing Division of Network Medicine | Cambridge | Massachusetts | 02138 | US |
| Ellinor, Patrick | Massachusetts General Hospital |  | Boston | Massachusetts | 02114 | US |
| Emery, Leslie | University of Washington |  | Seattle | Washington | 98195 | US |
| Erzurum, Serpil | Cleveland Clinic |  | Cleveland | Ohio | 44195 | US |
| Farber, Charles | University of Virginia |  | Charlottesville | Virginia | 22903 | US |
| Fingerlin, Tasha | National Jewish Health | Center for Genes, Environment and Health | Denver | Colorado | 80206 | US |
| Flickinger, Matthew | University of Michigan |  | Ann Arbor | Michigan | 48109 | US |
| Fornage, Myriam | University of Texas Health at Houston |  | Houston | Texas | 77225 | US |
| Franceschini, Nora | University of North Carolina | Epidemiology | Chapel Hill | North Carolina | 27599 | US |
| Frazar, Chris | University of Washington |  | Seattle | Washington | 98195 | US |
| Fu, Mao | University of Maryland |  | Baltimore | Maryland | 21201 | US |
| Fullerton, Stephanie M. | University of Washington |  | Seattle | Washington | 98195 | US |
| Fulton, Lucinda | Washington University in St Louis |  | St Louis | Missouri | 63130 | US |
| Gabriel, Stacey | Broad Institute |  | Cambridge | Massachusetts | 02142 | US |
| Gan, Weiniu | National Heart, Lung, and Blood Institute, National Institutes of Health |  | Bethesda | Maryland | 20892 | US |
| Gao, Shanshan | University of Colorado at Denver |  | Denver | Colorado | 80204 | US |
| Gao, Yan | University of Mississippi |  | Jackson | Mississippi | 38677 | US |
| Gass, Margery | Fred Hutchinson Cancer Research Center |  | Seattle | Washington | 98109 | US |
| Gelb, Bruce | Icahn School of Medicine at Mount Sinai |  | New York | New York | 10029 | US |
| Geng, Xiaoqi (Priscilla) | University of Michigan |  | Ann Arbor | Michigan | 48109 | US |
| Geraci, Mark | Indiana University | Medicine | Indianapolis | Indiana | 46202 | US |
| Germer, Soren | New York Genome Center |  | New York | New York | 10013 | US |
| Gerszten, Robert | Beth Israel Deaconess Medical Center |  | Boston | Massachusetts | 02215 | US |
| Ghosh, Auyon | Brigham & Women's Hospital |  | Boston | Massachusetts | 02115 | US |
| Gibbs, Richard | Baylor College of Medicine Human Genome Sequencing Center |  | Houston | Texas | 77030 | US |
| Gignoux, Chris | Stanford University |  | Stanford | California | 94305 | US |
| Gladwin, Mark | University of Pittsburgh |  | Pittsburgh | Pennsylvania | 15260 | US |
| Glahn, David | Boston Children's Hospital, Harvard Medical School | Department of Psychiatry | Boston | Massachusetts | 02115 | US |
| Gogarten, Stephanie | University of Washington |  | Seattle | Washington | 98195 | US |
| Gong, Da-Wei | University of Maryland |  | Baltimore | Maryland | 21201 | US |
| Goring, Harald | University of Texas Rio Grande Valley School of Medicine |  | San Antonio | Texas | 78229 | US |
| Graw, Sharon | University of Colorado Anschutz Medical Campus |  | Aurora | Colorado | 80045 | US |
| Gray, Kathryn J. | Mass General Brigham | Obstetrics and Gynecology | Boston | Massachusetts | 02115 | US |
| Grine, Daniel | University of Colorado at Denver |  | Denver | Colorado | 80204 | US |
| Gu, C. Charles | Washington University in St Louis |  | St Louis | Missouri | 63130 | US |
| Guan, Yue | University of Maryland |  | Baltimore | Maryland | 21201 | US |
| Guo, Xiuqing | Lundquist Institute |  | Torrance | California | 90502 | US |
| Gupta, Namrata | Broad Institute |  | Cambridge | Massachusetts | 02142 | US |
| Haas, David | Indiana University | OB/GYN | Indianapolis | Indiana | 46202 | US |
| Haessler, Jeff | Fred Hutchinson Cancer Research Center |  | Seattle | Washington | 98109 | US |
| Hall, Michael | University of Mississippi | Cardiology | Jackson | Mississippi | 39216 | US |
| Harris, Daniel | University of Maryland |  | Baltimore | Maryland | 21201 | US |
| Hawley, Nicola L. | Yale University | Department of Chronic Disease Epidemiology | New Haven | Connecticut | 06520 | US |
| He, Jiang | Tulane University |  | New Orleans | Louisiana | 70118 | US |
| Heavner, Ben | University of Washington | Biostatistics | Seattle | Washington | 98195 | US |
| Heckbert, Susan | University of Washington |  | Seattle | Washington | 98195 | US |
| Hernandez, Ryan | University of California, San Francisco |  | San Francisco | California | 94143 | US |
| Herrington, David | Wake Forest Baptist Health |  | Winston-Salem | North Carolina | 27157 | US |
| Hersh, Craig | Brigham & Women's Hospital | Channing Division of Network Medicine | Boston | Massachusetts | 02115 | US |
| Hidalgo, Bertha | University of Alabama |  | Birmingham | Alabama | 35487 | US |
| Hixson, James | University of Texas Health at Houston |  | Houston | Texas | 77225 | US |
| Hobbs, Brian | Brigham & Women's Hospital |  | Boston | Massachusetts | 02115 | US |
| Hokanson, John | University of Colorado at Denver |  | Denver | Colorado | 80204 | US |
| Hong, Elliott | University of Maryland |  | Baltimore | Maryland | 21201 | US |
| Hoth, Karin | University of Iowa |  | Iowa City | Iowa | 52242 | US |
| Hsiung, Chao (Agnes) | National Health Research Institute Taiwan | Institute of Population Health Sciences, NHRI | Miaoli County |  | 350 | TW |
| Hung, Yi-Jen | Tri-Service General Hospital National Defense Medical Center |  |  |  |  | TW |
| Huston, Haley | Blood Works Northwest |  | Seattle | Washington | 98104 | US |
| Hwu, Chii Min | Taichung Veterans General Hospital Taiwan |  | Taichung City |  | 407 | TW |
| Irvin, Marguerite Ryan | University of Alabama |  | Birmingham | Alabama | 35487 | US |
| Jackson, Rebecca | Oklahoma State University Medical Center | Internal Medicine, DIvision of Endocrinology, Diabetes and Metabolism | Columbus | Ohio | 43210 | US |
| Jain, Deepti | University of Washington |  | Seattle | Washington | 98195 | US |
| Jaquish, Cashell | National Heart, Lung, and Blood Institute, National Institutes of Health |  | Bethesda | Maryland | 20892 | US |
| Jhun, Min A | University of Michigan |  | Ann Arbor | Michigan | 48109 | US |
| Johnsen, Jill | Blood Works Northwest | Research Institute | Seattle | Washington | 98104 | US |
| Johnson, Andrew | National Heart, Lung, and Blood Institute, National Institutes of Health |  | Bethesda | Maryland | 20892 | US |
| Johnson, Craig | University of Washington |  | Seattle | Washington | 98195 | US |
| Johnston, Rich | Emory University |  | Atlanta | Georgia | 30322 | US |
| Jones, Kimberly | Johns Hopkins University |  | Baltimore | Maryland | 21218 | US |
| Kang, Hyun Min | University of Michigan | Biostatistics | Ann Arbor | Michigan | 48109 | US |
| Kaplan, Robert | Albert Einstein College of Medicine |  | New York | New York | 10461 | US |
| Kardia, Sharon | University of Michigan |  | Ann Arbor | Michigan | 48109 | US |
| Kathiresan, Sekar | Broad Institute |  | Cambridge | Massachusetts | 02142 | US |
| Kelly, Shannon | Vitalant Research Institute |  | San Francisco | California | 94118 | US |
| Kenny, Eimear | Icahn School of Medicine at Mount Sinai |  | New York | New York | 10029 | US |
| Kessler, Michael | University of Maryland |  | Baltimore | Maryland | 21201 | US |
| Khan, Alyna | University of Washington |  | Seattle | Washington | 98195 | US |
| Kim, Wonji | Harvard University |  | Cambridge | Massachusetts | 02138 | US |
| Kinney, Greg | University of Colorado at Denver | Epidemiology | Aurora | Colorado | 80045 | US |
| Konkle, Barbara | Blood Works Northwest |  | Seattle | Washington | 98104 | US |
| Kooperberg, Charles | Fred Hutchinson Cancer Research Center |  | Seattle | Washington | 98109 | US |
| Kramer, Holly | Loyola University | Public Health Sciences | Maywood | Illinois | 60153 | US |
| Lange, Christoph | Harvard School of Public Health | Biostats | Boston | Massachusetts | 02115 | US |
| Lange, Ethan | University of Colorado at Denver |  | Denver | Colorado | 80204 | US |
| Lange, Leslie | University of Colorado at Denver |  | Denver | Colorado | 80204 | US |
| Laurie, Cathy | University of Washington |  | Seattle | Washington | 98195 | US |
| Laurie, Cecelia | University of Washington |  | Seattle | Washington | 98195 | US |
| LeBoff, Meryl | Brigham & Women's Hospital |  | Boston | Massachusetts | 02115 | US |
| Lee, Jiwon | Brigham & Women's Hospital |  | Boston | Massachusetts | 02115 | US |
| Lee, Seunggeun Shawn | University of Michigan |  | Ann Arbor | Michigan | 48109 | US |
| Lee, Wen-Jane | Taichung Veterans General Hospital Taiwan |  | Taichung City |  | 407 | TW |
| LeFaive, Jonathon | University of Michigan |  | Ann Arbor | Michigan | 48109 | US |
| Levine, David | University of Washington |  | Seattle | Washington | 98195 | US |
| Levy, Dan | National Heart, Lung, and Blood Institute, National Institutes of Health |  | Bethesda | Maryland | 20892 | US |
| Lewis, Joshua | University of Maryland |  | Baltimore | Maryland | 21201 | US |
| Li, Xiaohui | Lundquist Institute |  | Torrance | California | 90502 | US |
| Li, Yun | University of North Carolina |  | Chapel Hill | North Carolina | 27599 | US |
| Lin, Henry | Lundquist Institute |  | Torrance | California | 90502 | US |
| Lin, Honghuang | Boston University |  | Boston | Massachusetts | 02215 | US |
| Lin, Keng Han | University of Michigan |  | Ann Arbor | Michigan | 48109 | US |
| Lin, Xihong | Harvard School of Public Health |  | Boston | Massachusetts | 02115 | US |
| Liu, Simin | Brown University | Epidemiology and Medicine | Providence | Rhode Island | 02912 | US |
| Liu, Yongmei | Duke University | Cardiology | Durham | North Carolina | 27708 | US |
| Liu, Yu | Stanford University | Cardiovascular Institute | Stanford | California | 94305 | US |
| Loos, Ruth J.F. | Icahn School of Medicine at Mount Sinai | The Charles Bronfman Institute for Personalized Medicine | New York | New York | 10029 | US |
| Lubitz, Steven | Massachusetts General Hospital |  | Boston | Massachusetts | 02114 | US |
| Lunetta, Kathryn | Boston University |  | Boston | Massachusetts | 02215 | US |
| Luo, James | National Heart, Lung, and Blood Institute, National Institutes of Health |  | Bethesda | Maryland | 20892 | US |
| Magalan, Ulysses | Oklahoma State University Medical Center |  |  |  |  | US |
| Mahaney, Michael | University of Texas Rio Grande Valley School of Medicine |  | Brownsville | Texas | 78520 | US |
| Make, Barry | Johns Hopkins University |  | Baltimore | Maryland | 21218 | US |
| Manichaikul, Ani | University of Virginia |  | Charlottesville | Virginia | 22903 | US |
| Manson, JoAnn | Brigham & Women's Hospital |  | Boston | Massachusetts | 02115 | US |
| Margolin, Lauren | Broad Institute |  | Cambridge | Massachusetts | 02142 | US |
| Martin, Lisa | George Washington University |  | Washington | District of Columbia | 20052 | US |
| Mathai, Susan | University of Colorado at Denver |  | Denver | Colorado | 80204 | US |
| Mathias, Rasika | Johns Hopkins University |  | Baltimore | Maryland | 21218 | US |
| May, Susanne | University of Washington | Biostatistics | Seattle | Washington | 98195 | US |
| McArdle, Patrick | University of Maryland |  | Baltimore | Maryland | 21201 | US |
| McDonald, Merry-Lynn | University of Alabama |  | Birmingham | Alabama | 35487 | US |
| McFarland, Sean | Harvard University |  | Cambridge | Massachusetts | 02138 | US |
| McGarvey, Stephen | Brown University |  | Providence | Rhode Island | 02912 | US |
| McGoldrick, Daniel | University of Washington |  | Seattle | Washington | 98195 | US |
| McHugh, Caitlin | University of Washington | Biostatistics | Seattle | Washington | 98195 | US |
| McNeil, Becky | RTI International |  |  |  |  | US |
| Mei, Hao | University of Mississippi |  | Jackson | Mississippi | 38677 | US |
| Mestroni, Luisa | University of Colorado Anschutz Medical Campus |  | Aurora | Colorado | 80045 | US |
| Meyers, Deborah A | University of Arizona |  | Tucson | Arizona | 85721 | US |
| Mignot, Emmanuel | Stanford University | Center For Sleep Sciences and Medicine | Palo Alto | California | 94304 | US |
| Mikulla, Julie | National Heart, Lung, and Blood Institute, National Institutes of Health |  | Bethesda | Maryland | 20892 | US |
| Min, Nancy | University of Mississippi |  | Jackson | Mississippi | 38677 | US |
| Minear, Mollie | National Heart, Lung, and Blood Institute, National Institutes of Health |  | Bethesda | Maryland | 20892 | US |
| Minster, Ryan L | University of Pittsburgh |  | Pittsburgh | Pennsylvania | 15260 | US |
| Mitchell, Braxton D. | University of Maryland |  | Baltimore | Maryland | 21201 | US |
| Moll, Matt | Brigham & Women's Hospital | Medicine | Boston | Massachusetts | 02115 | US |
| Montasser, May E. | University of Maryland |  | Baltimore | Maryland | 21201 | US |
| Montgomery, Courtney | Oklahoma Medical Research Foundation | Genes and Human Disease | Oklahoma City | Oklahoma | 73104 | US |
| Moscati, Arden | Icahn School of Medicine at Mount Sinai |  | New York | New York | 10029 | US |
| Musani, Solomon | University of Mississippi | Medicine | Jackson | Mississippi | 38677 | US |
| Mwasongwe, Stanford | University of Mississippi |  | Jackson | Mississippi | 38677 | US |
| Mychaleckyj, Josyf C | University of Virginia |  | Charlottesville | Virginia | 22903 | US |
| Nadkarni, Girish | Icahn School of Medicine at Mount Sinai |  | New York | New York | 10029 | US |
| Naik, Rakhi | Johns Hopkins University |  | Baltimore | Maryland | 21218 | US |
| Naseri, Take | Ministry of Health, Government of Samoa |  | Apia |  |  | WS |
| Natarajan, Pradeep | Broad Institute |  | Cambridge | Massachusetts | 02142 | US |
| Nekhai, Sergei | Howard University |  | Washington | District of Columbia | 20059 | US |
| Nelson, Sarah C. | University of Washington | Biostatistics | Seattle | Washington | 98195 | US |
| Neltner, Bonnie | University of Colorado at Denver |  | Denver | Colorado | 80204 | US |
| Nickerson, Deborah | University of Washington |  | Seattle | Washington | 98195 | US |
| North, Kari | University of North Carolina |  | Chapel Hill | North Carolina | 27599 | US |
| O'Connell, Jeff | University of Maryland |  | Balitmore | Maryland | 21201 | US |
| O'Connor, Tim | University of Maryland |  | Baltimore | Maryland | 21201 | US |
| Ochs-Balcom, Heather | University at Buffalo |  | Buffalo | New York | 14260 | US |
| Pack, Allan | University of Pennsylvania | Division of Sleep Medicine/Department of Medicine | Philadelphia | Pennsylvania | 19104-3403 | US |
| Paik, David T. | Stanford University | Stanford Cardiovascular Institute | Stanford | California | 94305 | US |
| Palmer, Nicholette | Wake Forest Baptist Health | Biochemistry | Winston-Salem | North Carolina | 27157 | US |
| Pankow, James | University of Minnesota |  | Minneapolis | Minnesota | 55455 | US |
| Papanicolaou, George | National Heart, Lung, and Blood Institute, National Institutes of Health |  | Bethesda | Maryland | 20892 | US |
| Parker, Cora | RTI International | Biostatistics and Epidemiology Division | Research Triangle Park | North Carolina | 27709-2194 | US |
| Parsa, Afshin | University of Maryland |  | Baltimore | Maryland | 21201 | US |
| Peralta, Juan Manuel | University of Texas Rio Grande Valley School of Medicine |  | Edinburg | Texas | 78539 | US |
| Perez, Marco | Stanford University |  | Stanford | California | 94305 | US |
| Perry, James | University of Maryland |  | Baltimore | Maryland | 21201 | US |
| Peters, Ulrike | Fred Hutchinson Cancer Research Center |  | Seattle | Washington | 98109 | US |
| Peyser, Patricia | University of Michigan |  | Ann Arbor | Michigan | 48109 | US |
| Phillips, Lawrence S | Emory University |  | Atlanta | Georgia | 30322 | US |
| Pollin, Toni | University of Maryland |  | Baltimore | Maryland | 21201 | US |
| Post, Wendy | Johns Hopkins University | Cardiology/Medicine | Baltimore | Maryland | 21218 | US |
| Powers Becker, Julia | University of Colorado at Denver | Medicine | Denver | Colorado | 80204 | US |
| Preethi Boorgula, Meher | University of Colorado at Denver |  | Denver | Colorado | 80204 | US |
| Preuss, Michael | Icahn School of Medicine at Mount Sinai |  | New York | New York | 10029 | US |
| Psaty, Bruce | University of Washington |  | Seattle | Washington | 98195 | US |
| Qasba, Pankaj | National Heart, Lung, and Blood Institute, National Institutes of Health |  | Bethesda | Maryland | 20892 | US |
| Qiao, Dandi | Brigham & Women's Hospital |  | Boston | Massachusetts | 02115 | US |
| Qin, Zhaohui | Emory University |  | Atlanta | Georgia | 30322 | US |
| Rafaels, Nicholas | University of Colorado at Denver |  | Denver | Colorado | 80045 | US |
| Raffield, Laura | University of North Carolina | Genetics | Chapel Hill | North Carolina | 27599 | US |
| Ramachandran , Vasan S. | Boston University |  | Boston | Massachusetts | 02215 | US |
| Rao, D.C. | Washington University in St Louis |  | St Louis | Missouri | 63130 | US |
| Rasmussen-Torvik, Laura | Northwestern University |  | Chicago | Illinois | 60208 | US |
| Ratan, Aakrosh | University of Virginia |  | Charlottesville | Virginia | 22903 | US |
| Redline, Susan | Brigham & Women's Hospital |  | Boston | Massachusetts | 02115 | US |
| Reed, Robert | University of Maryland |  | Baltimore | Maryland | 21201 | US |
| Regan, Elizabeth | National Jewish Health |  | Denver | Colorado | 80206 | US |
| Reiner, Alex | Fred Hutchinson Cancer Research Center, University of Washington |  | Seattle | Washington | 98109 | US |
| Reupena, Muagututi‘a Sefuiva | Lutia I Puava Ae Mapu I Fagalele |  | Apia |  |  | WS |
| Rice, Ken | University of Washington |  | Seattle | Washington | 98195 | US |
| Rich, Stephen | University of Virginia |  | Charlottesville | Virginia | 22903 | US |
| Roden, Dan | Vanderbilt University | Medicine, Pharmacology, Biomedicla Informatics | Nashville | Tennessee | 37235 | US |
| Roselli, Carolina | Broad Institute |  | Cambridge | Massachusetts | 02142 | US |
| Rotter, Jerome | Lundquist Institute |  | Torrance | California | 90502 | US |
| Ruczinski, Ingo | Johns Hopkins University |  | Baltimore | Maryland | 21218 | US |
| Russell, Pamela | University of Colorado at Denver |  | Denver | Colorado | 80204 | US |
| Ruuska, Sarah | Blood Works Northwest |  | Seattle | Washington | 98104 | US |
| Ryan, Kathleen | University of Maryland |  | Baltimore | Maryland | 21201 | US |
| Sabino, Ester Cerdeira | Universidade de Sao Paulo | Faculdade de Medicina | Sao Paulo |  | 01310000 | BR |
| Saleheen, Danish | Columbia University |  | New York | New York | 10027 | US |
| Salimi, Shabnam | University of Maryland |  | Baltimore | Maryland | 21201 | US |
| Salzberg, Steven | Johns Hopkins University |  | Baltimore | Maryland | 21218 | US |
| Sandow, Kevin | Lundquist Institute | TGPS | Torrance | California | 90502 | US |
| Sankaran, Vijay G. | Harvard University | Division of Hematology/Oncology | Cambridge | Massachusetts | 02138 | US |
| Scheller, Christopher | University of Michigan |  | Ann Arbor | Michigan | 48109 | US |
| Schmidt, Ellen | University of Michigan |  | Ann Arbor | Michigan | 48109 | US |
| Schwander, Karen | Washington University in St Louis |  | St Louis | Missouri | 63130 | US |
| Schwartz, David | University of Colorado at Denver |  | Denver | Colorado | 80204 | US |
| Sciurba, Frank | University of Pittsburgh |  | Pittsburgh | Pennsylvania | 15260 | US |
| Seidman, Christine | Harvard Medical School | Genetics | Boston | Massachusetts | 02115 | US |
| Seidman, Jonathan | Harvard Medical School |  | Boston | Massachusetts | 02115 | US |
| Sheehan, Vivien | Baylor College of Medicine | Pediatrics | Atlanta | Georgia | 30307 | US |
| Sherman, Stephanie L. | Emory University | Human Genetics | Atlanta | Georgia | 30322 | US |
| Shetty, Amol | University of Maryland |  | Baltimore | Maryland | 21201 | US |
| Shetty, Aniket | University of Colorado at Denver |  | Denver | Colorado | 80204 | US |
| Sheu, Wayne Hui-Heng | Taichung Veterans General Hospital Taiwan |  | Taichung City |  | 407 | TW |
| Shoemaker, M. Benjamin | Vanderbilt University | Medicine/Cardiology | Nashville | Tennessee | 37235 | US |
| Silver, Brian | UMass Memorial Medical Center |  | Worcester | Massachusetts | 01655 | US |
| Silverman, Edwin | Brigham & Women's Hospital |  | Boston | Massachusetts | 02115 | US |
| Smith, Jennifer | University of Michigan |  | Ann Arbor | Michigan | 48109 | US |
| Smith, Josh | University of Washington |  | Seattle | Washington | 98195 | US |
| Smith, Nicholas | University of Washington | Epidemiology | Seattle | Washington | 98195 | US |
| Smith, Tanja | New York Genome Center |  | New York | New York | 10013 | US |
| Smoller, Sylvia | Albert Einstein College of Medicine |  | New York | New York | 10461 | US |
| Snively, Beverly | Wake Forest Baptist Health | Biostatistical Sciences | Winston-Salem | North Carolina | 27157 | US |
| Snyder, Michael | Stanford University |  | Stanford | California | 94305 | US |
| Sofer, Tamar | Brigham & Women's Hospital |  | Boston | Massachusetts | 02115 | US |
| Sotoodehnia, Nona | University of Washington |  | Seattle | Washington | 98195 | US |
| Stilp, Adrienne M. | University of Washington |  | Seattle | Washington | 98195 | US |
| Storm, Garrett | University of Colorado at Denver |  | Denver | Colorado | 80204 | US |
| Streeten, Elizabeth | University of Maryland |  | Baltimore | Maryland | 21201 | US |
| Su, Jessica Lasky | Brigham & Women's Hospital |  | Boston | Massachusetts | 02115 | US |
| Sung, Yun Ju | Washington University in St Louis |  | St Louis | Missouri | 63130 | US |
| Sylvia, Jody | Brigham & Women's Hospital |  | Boston | Massachusetts | 02115 | US |
| Szpiro, Adam | University of Washington |  | Seattle | Washington | 98195 | US |
| Sztalryd, Carole | University of Maryland |  | Baltimore | Maryland | 21201 | US |
| Taliun, Daniel | University of Michigan |  | Ann Arbor | Michigan | 48109 | US |
| Tang, Hua | Stanford University | Genetics | Stanford | California | 94305 | US |
| Taub, Margaret | Johns Hopkins University |  | Baltimore | Maryland | 21218 | US |
| Taylor, Kent D. | Lundquist Institute | Institute for Translational Genomics and Populations Sciences | Torrance | California | 90502 | US |
| Taylor, Matthew | University of Colorado Anschutz Medical Campus |  | Aurora | Colorado | 80045 | US |
| Taylor, Simeon | University of Maryland |  | Baltimore | Maryland | 21201 | US |
| Telen, Marilyn | Duke University |  | Durham | North Carolina | 27708 | US |
| Thornton, Timothy A. | University of Washington |  | Seattle | Washington | 98195 | US |
| Threlkeld, Machiko | University of Washington | University of Washington, Department of Genome Sciences | Seattle | Washington | 98195 | US |
| Tinker, Lesley | Fred Hutchinson Cancer Research Center |  | Seattle | Washington | 98109 | US |
| Tirschwell, David | University of Washington |  | Seattle | Washington | 98195 | US |
| Tishkoff, Sarah | University of Pennsylvania | Genetics | Philadelphia | Pennsylvania | 19104 | US |
| Tiwari, Hemant | University of Alabama | Biostatistics | Birmingham | Alabama | 35487 | US |
| Tong, Catherine | University of Washington | Department of Biostatistics | Seattle | Washington | 98195 | US |
| Tracy, Russell | University of Vermont | Pathology & Laboratory Medicine | Burlington | Vermont | 05405 | US |
| Tsai, Michael | University of Minnesota |  | Minneapolis | Minnesota | 55455 | US |
| Vaidya, Dhananjay | Johns Hopkins University |  | Baltimore | Maryland | 21218 | US |
| Van Den Berg, David | University of Southern California | USC Methylation Characterization Center | University of Southern California | California | 90033 | US |
| VandeHaar, Peter | University of Michigan |  | Ann Arbor | Michigan | 48109 | US |
| Vrieze, Scott | University of Minnesota |  | Minneapolis | Minnesota | 55455 | US |
| Walker, Tarik | University of Colorado at Denver |  | Denver | Colorado | 80204 | US |
| Wallace, Robert | University of Iowa |  | Iowa City | Iowa | 52242 | US |
| Walts, Avram | University of Colorado at Denver |  | Denver | Colorado | 80204 | US |
| Wang, Fei Fei | University of Washington |  | Seattle | Washington | 98195 | US |
| Wang, Heming | Brigham & Women's Hospital, Mass General Brigham |  | Boston | Massachusetts | 02115 | US |
| Watson, Karol | University of California, Los Angeles |  | Los Angeles | California | 90095 | US |
| Weeks, Daniel E. | University of Pittsburgh |  | Pittsburgh | Pennsylvania | 15260 | US |
| Weir, Bruce | University of Washington |  | Seattle | Washington | 98195 | US |
| Weiss, Scott | Brigham & Women's Hospital |  | Boston | Massachusetts | 02115 | US |
| Weng, Lu-Chen | Massachusetts General Hospital |  | Boston | Massachusetts | 02114 | US |
| Wessel, Jennifer | Indiana University | Epidemiology | Indianapolis | Indiana | 46202 | US |
| Willer, Cristen | University of Michigan | Internal Medicine | Ann Arbor | Michigan | 48109 | US |
| Williams, Kayleen | University of Washington | Biostatistics | Seattle | Washington | 98195 | US |
| Williams, L. Keoki | Henry Ford Health System |  | Detroit | Michigan | 48202 | US |
| Wilson, Carla | Brigham & Women's Hospital |  | Boston | Massachusetts | 02115 | US |
| Wilson, James | Beth Israel Deaconess Medical Center | Cardiology | Cambridge | Massachusetts | 02139 | US |
| Wong, Quenna | University of Washington |  | Seattle | Washington | 98195 | US |
| Wu, Joseph | Stanford University | Stanford Cardiovascular Institute | Stanford | California | 94305 | US |
| Xu, Huichun | University of Maryland |  | Baltimore | Maryland | 21201 | US |
| Yanek, Lisa | Johns Hopkins University |  | Baltimore | Maryland | 21218 | US |
| Yang, Ivana | University of Colorado at Denver |  | Denver | Colorado | 80204 | US |
| Yang, Rongze | University of Maryland |  | Baltimore | Maryland | 21201 | US |
| Zaghloul, Norann | University of Maryland |  | Baltimore | Maryland | 21201 | US |
| Zekavat, Maryam | Broad Institute |  | Cambridge | Massachusetts | 02142 | US |
| Zhang, Yingze | University of Pittsburgh | Medicine | Pittsburgh | Pennsylvania | 15260 | US |
| Zhao, Snow Xueyan | National Jewish Health |  | Denver | Colorado | 80206 | US |
| Zhao, Wei | University of Michigan | Department of Epidemiology | Ann Arbor | Michigan | 48109 | US |
| Zhi, Degui | University of Texas Health at Houston |  | Houston | Texas | 77225 | US |
| Zhou, Xiang | University of Michigan |  | Ann Arbor | Michigan | 48109 | US |
| Zhu, Xiaofeng | Case Western Reserve University | Department of Population and Quantitative Health Sciences | Cleveland | Ohio | 44106 | US |
| Zody, Michael | New York Genome Center |  | New York | New York | 10013 | US |
| Zoellner, Sebastian | University of Michigan |  | Ann Arbor | Michigan | 48109 | US |
